# Supplementary material for: Does cranberry extract reduce antibiotic use for symptoms of acute uncomplicated urinary tract infections (CUTI)? A feasibility randomised trial
Source: BMJ Open. 2021 Feb 22;11(2):e046791. doi: 10.1136/bmjopen-2020-046791 (PMC7903114; doi:10.1136/bmjopen-2020-046791)
Supplement: Supplementary data [file bmjopen-2020-046791supp001.pdf]

Supplementary Figures

Kaplan Meier survival plot showing the duration of symptoms rated moderately bad or worse by group

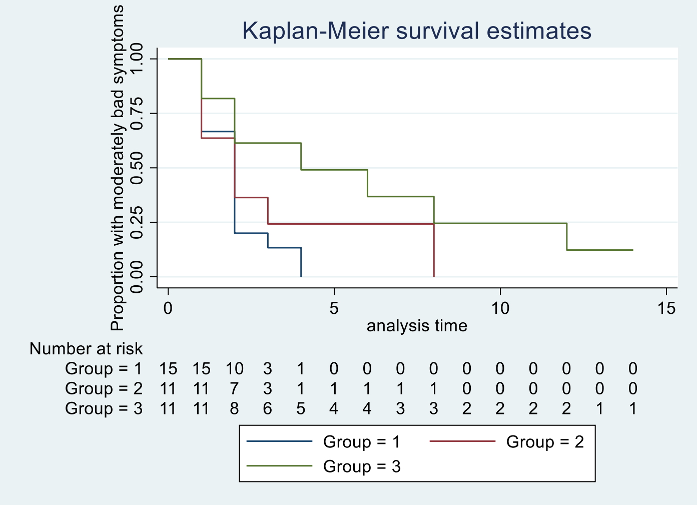

Key: Group 1: Immediate Antibiotics alone; Group 2: Immediate Antibiotics and Immediate Cranberry; Group 3: Immediate Cranberry and Delayed Antibiotics

Kaplan Meier survival plot showing the time to feeling fully recovered by group

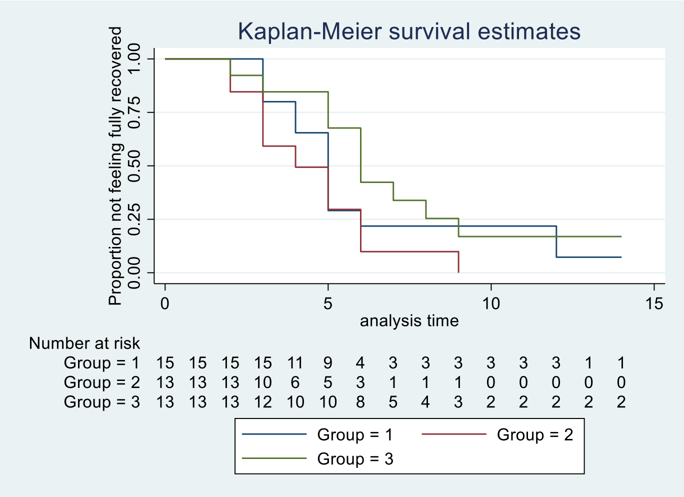

Key: Group 1: Immediate Antibiotics alone; Group 2: Immediate Antibiotics and Immediate Cranberry; Group 3: Immediate Cranberry and Delayed Antibiotics
