# Supplementary material for: Does cranberry extract reduce antibiotic use for symptoms of acute uncomplicated urinary tract infections (CUTI)? A feasibility randomised trial
Source: BMJ Open. 2021 Feb 22;11(2):e046791. doi: 10.1136/bmjopen-2020-046791 (PMC7903114; doi:10.1136/bmjopen-2020-046791)
Supplement: Supplementary data [file bmjopen-2020-046791supp002.pdf]

BMJ Publishing Group Limited (BMJ) disclaims all liability and responsibility arising from any reliance placed on this supplemental material which has been supplied by the author(s)

Supplementary file 1 – Estimate of the difference in proportions of adverse events between groups

	Proportion (%) of participants experiencing an adverse event at 2 weeks [risk ratio (95% CI)]	Proportion (%) of participants experiencing an adverse event at 4 weeks [risk ratio (95% CI)]
**Group 1** (Control) n=15	1/15 (6.7%)<br><br>Missing: 0/15	2/15 (13.3%)<br><br>Missing: 0/15
**Group 2** n=15	1/13 (7.7%)<br>[1.2 (0.1 to 16.7)]<br><br>Missing: 2/15	1/13 (7.7%)<br>[0.6 (0.1 to 5.7)]<br><br>Missing: 2/15
**Group 3** n=16	3/13 (23.1%)<br>[3.5 (0.4 to 29.4)]<br><br>Missing: 3/16	3/13 (23.1%)<br>[1.7 (0.3 to 8.8)]<br><br>Missing: 3/16

Group 1 – Immediate antibiotics alone (control); Group 2 – Immediate antibiotics and immediate cranberry; Group 3 – Immediate cranberry and delayed antibiotics. Group 2 and group 3 were compared with Group 1 (controls).
