# Supplementary material for: Does cranberry extract reduce antibiotic use for symptoms of acute uncomplicated urinary tract infections (CUTI)? A feasibility randomised trial
Source: BMJ Open. 2021 Feb 22;11(2):e046791. doi: 10.1136/bmjopen-2020-046791 (PMC7903114; doi:10.1136/bmjopen-2020-046791)
Supplement: Author's manuscript [file bmjopen-2020-046791.draft_revisions.pdf]

BMJ Open

BMJ Open is committed to open peer review. As part of this commitment we make the peer review history of every article we publish publicly available.

When an article is published we post the peer reviewers' comments and the authors' responses online. We also post the versions of the paper that were used during peer review. These are the versions that the peer review comments apply to.

The versions of the paper that follow are the versions that were submitted during the peer review process. They are not the versions of record or the final published versions. They should not be cited or distributed as the published version of this manuscript.

BMJ Open is an open access journal and the full, final, typeset and author-corrected version of record of the manuscript is available on our site with no access controls, subscription charges or pay-per-view fees (http://bmjopen.bmj.com).

If you have any questions on BMJ Open's open peer review process please email info.bmjopen@bmj.com

BMJ Open

# Does cranberry extract reduce antibiotic use for symptoms of acute uncomplicated urinary tract infections (CUTI)? A feasibility randomised trial.

| | |
|---|---|
| Journal: | *BMJ Open* |
| Manuscript ID | bmjopen-2020-046791 |
| Article Type: | Original research |
| Date Submitted by the Author: | 09-Nov-2020 |
| Complete List of Authors: | Gbinigie, Oghenekome; University of Oxford, Nuffield Department of Primary Care Health Sciences<br>Allen, Julie; University of Oxford, Nuffield Department of Primary Care Health Sciences<br>Williams, Nicola; Nuffield Department of Primary Care Health Sciences, University of Oxford<br>Moore, Michael; University of Southampton Medical School, Primary Care Medical Group<br>Hay, Alastair; University of Bristol, Centre for Academic Primary Care<br>Heneghan, Carl; Oxford University, Primary Health Care<br>Boylan, Anne-Marie; Oxford University, Nuffield Department of Primary Care Health Sciences<br>Butler, Christopher C.; University of Oxford, |
| Keywords: | Urinary tract infections < UROLOGY, PRIMARY CARE, Clinical trials < THERAPEUTICS |
| | |

SCHOLARONE™
Manuscripts

*I, the Submitting Author has the right to grant and does grant on behalf of all authors of the Work (as defined in the below author licence), an exclusive licence and/or a non-exclusive licence for contributions from authors who are: i) UK Crown employees; ii) where BMJ has agreed a CC-BY licence shall apply, and/or iii) in accordance with the terms applicable for US Federal Government officers or employees acting as part of their official duties; on a worldwide, perpetual, irrevocable, royalty-free basis to BMJ Publishing Group Ltd ("BMJ") its licensees and where the relevant Journal is co-owned by BMJ to the co-owners of the Journal, to publish the Work in this journal and any other BMJ products and to exploit all rights, as set out in our licence.*

*The Submitting Author accepts and understands that any supply made under these terms is made by BMJ to the Submitting Author unless you are acting as an employee on behalf of your employer or a postgraduate student of an affiliated institution which is paying any applicable article publishing charge ("APC") for Open Access articles. Where the Submitting Author wishes to make the Work available on an Open Access basis (and intends to pay the relevant APC), the terms of reuse of such Open Access shall be governed by a Creative Commons licence – details of these licences and which Creative Commons licence will apply to this Work are set out in our licence referred to above.*

*Other than as permitted in any relevant BMJ Author's Self Archiving Policies, I confirm this Work has not been accepted for publication elsewhere, is not being considered for publication elsewhere and does not duplicate material already published. I confirm all authors consent to publication of this Work and authorise the granting of this licence.*

For peer review only

**Title:** Does cranberry extract reduce antibiotic use for symptoms of acute uncomplicated urinary tract infections (CUTI)? A feasibility randomised trial.

**Authors' names:** Oghenekome A Gbinigie[1] (0000-0002-2963-4491), Julie Allen[1], Nicola Williams[1], Michael Moore[2], Alastair D Hay[3], Carl J Heneghan[1], Anne-Marie Boylan[1], Chris C Butler[1]

**Authors' addresses:**

[1] Nuffield Department of Primary Care Health Sciences, University of Oxford, Radcliffe Observatory Quarter, Woodstock Road, Oxford, OX2 6GG.

[2] Primary Care and Population Sciences, University of Southampton, SO16 5ST.

[3]Centre for Academic Primary Care, Population Health Sciences: Bristol Medical School, University of Bristol, BS8 2PS.

**Corresponding author:**

Dr Oghenekome Gbinigie

Nuffield Department of Primary Care Health Sciences, University of Oxford, Radcliffe Observatory Quarter, Woodstock Road, Oxford, OX2 6GG.

Email address: oghenekome.gbinigie@phc.ox.ac.uk

**Word count:** 4177

**Keywords:** Urinary Tract Infection, Vaccinium macrocarpon, General Practice, Primary Health Care, Clinical Trial



For peer review only - http://bmjopen.bmj.com/site/about/guidelines.xhtml

**Abstract**

**Objectives:** To determine the feasibility of conducting a randomised trial of the effectiveness of cranberry extract in reducing antibiotic use by women with symptoms of acute, uncomplicated urinary tract infection (UTI).

**Design:** Open-label feasibility randomised trial.

**Setting:** Four general practices in Oxfordshire

**Participants:** Women aged 18 years and above presenting to general practice with symptoms of acute, uncomplicated UTI.

**Interventions:** Women were randomly assigned in a 1:1:1 ratio to: 1) Immediate antibiotics alone; 2) Immediate antibiotics and immediate cranberry capsules for up to 7 days; or 3) Immediate cranberry capsules and delayed antibiotics for self-initiation in case of non-improvement or worsening of symptoms.

**Primary and secondary outcomes measures:** The primary outcome measures were: rate of recruitment of participants; numbers lost to follow-up; proportion of electronic diaries completed by participants; and acceptability of the intervention and study procedures to participants and recruiters. Secondary outcomes included an exploration of differences in symptom burden and antibiotic use between groups.

**Results:** Four GP practices (100% of target) were recruited between 1$^{st}$ July and 2$^{nd}$ December 2019, with nine study participants recruited per month on average. 68.7% (46/67) of eligible participants were randomised (target 45) with a mean age of 48.4 years (SD 19.9, range 18 to 81). 89.1% (41/46) of diaries contained some participant entered data, and 69.6% (32/46) were fully complete. Three participants (6.5%) were lost to follow-up and two (4.4%) withdrew. Of women randomly assigned to take antibiotics alone (controls), one third of respondents reported consuming cranberry products (33.3%, 4/12).




For peer review only - http://bmjopen.bmj.com/site/about/guidelines.xhtml

**Conclusions:** It appears feasible to conduct a randomised trial of the use of cranberry extract in the treatment of acute, uncomplicated UTI in general practice.

**Trial registration:** ISRCTN registry, ID: 10399299. Registered prospectively on 24th January 2019

**Strengths and limitations of this study**

- This feasibility trial addresses a novel use of cranberry extract in the treatment of *acute*, rather than prevention of *recurrent*, urinary tract infection.
- A pragmatic, open-label randomised trial design was employed.
- In keeping with pragmatic trials, no placebo control was used.

For peer review only

## Introduction

In light of rising levels of antimicrobial resistance, there has been growing interest in the use of non-antibiotic treatments for common bacterial infections, such as urinary tract infections (UTIs). UTIs are one of the commonest bacterial infections seen in primary care[1], and are nearly always treated with antibiotics.[2] Trials have evaluated non-antibiotic treatments for acute uncomplicated UTIs in primary care, [3-7] and use of ibuprofen has enabled some women to manage UTIs without taking antibiotics, albeit with a greater symptom burden compared with antibiotic treatment, and more cases of pyelonephritis.[3 6 7]

Cranberry has been extensively evaluated as a potential treatment for recurrent UTI, with mixed results. Up to 27% of women report using cranberry to help treat the symptoms of an acute UTI. [8] Despite this, there is a lack of trials assessing whether cranberry can be used to treat the symptoms of *acute* UTIs, alone, or in combination with antibiotics. There is some mechanistic data to support this approach; urine collected within 6 hours of consumption of cranberry powder containing standardized amounts of Proanthocyanidins (PAC) has been shown to exert an anti-adhesion effect against Escherichia coli *in vitro*, in a dose-dependent fashion.[9] PAC with type A linkages, or the breakdown products after digestion in the gut, is believed to be the active ingredient in cranberry.[10]

The primary objective of this feasibility trial was therefore to determine the feasibility of conducting a randomised clinical trial to determine the effectiveness of cranberry extract in reducing antibiotic use by women with symptoms of acute, uncomplicated urinary tract infection (UTI) in general practice.



For peer review only - http://bmjopen.bmj.com/site/about/guidelines.xhtml

## Methods

Trial Design and Participants

This was an open-label, pragmatic, feasibility trial with randomisation in a 1:1:1 ratio between treatment groups in UK primary care. The eligibility criteria have been described in detail elsewhere.[11] In brief, women aged 18 years and above with symptoms of acute, uncomplicated UTI, to whom their healthcare practitioner would normally give a prescription for immediate antibiotics, and presenting to one of four participating General Practitioner (GP) practices in Oxfordshire, were eligible. Prospective participants had to be prepared to accept a delayed antibiotic prescription; that is, an antibiotic prescription with advice not to take the antibiotics unless their symptoms did not improve, or worsened. Women were excluded if they had signs of upper or complicated UTI, if they had received antibiotics in the preceding 7 days, if they had a symptom duration of 7 days or more, or if they regularly consumed cranberry products on five or more days per week.

Participants were emailed a link to an electronic symptom diary daily for 2 weeks. They were asked to rate a number of symptoms on a scale of 0 to 50, and to record any UTI treatments (study interventions or otherwise) that they were taking. The scale used was an adaptation of a validated 7-point Likert scale developed by Watson *et al* [12]:

0 = Normal/not affected

1–9 = Very little problem

10–19 = Slight problem

20–29 = Moderately bad

30–39 = Bad

40–49 = Very bad

50 = As bad as it could be



For peer review only - http://bmjopen.bmj.com/site/about/guidelines.xhtml

A follow-up phone call took place at 2 weeks to encourage diary completion and obtain a minimum data set from participants whose diaries were incomplete. Women were invited for interview at a later date if they had provided consent at the point of trial recruitment to receive information about the interview study.

Interventions

Women in Group 1 (control) received a prescription for first-line immediate antibiotics alone. In keeping with pragmatic trial design, the antibiotic prescribed and the duration was at the discretion of the recruiting clinician. In the UK, the usual recommended first-line treatment for acute uncomplicated UTI is a 3 day course of Nitrofurantoin.[13] Women assigned to Group 2 received an immediate prescription for first-line antibiotics and a course of cranberry capsules to be taken alongside antibiotics until symptom resolution, or up to a maximum of seven days. The cranberry capsules (Redicran) were supplied free to the study by Indena S.p.A. This company had no other involvement with the trial. Each capsule contained 18 mg of PAC. Women were advised to take two capsules twice daily at 12 hour intervals, making a total of 72 mg of PAC daily. Ex-vivo studies suggest that consumption of 72 mg PAC has anti-adhesion activity against Uropathogenic Escherichia coli (UPEC). [9] Women in Group 3 were advised to take immediate cranberry capsules (Redicran) for up to seven days. They also received a delayed antibiotic prescription to be used if their symptoms did not improve within 3-5 days, or if their symptoms worsened.

Outcomes

The primary outcomes were feasibility measures,[11] namely, the rate of recruitment of participants, the number of participants lost to follow-up, and the acceptability of trial processes and the quality of data capture using electronic symptom diaries. These data can



For peer review only - http://bmjopen.bmj.com/site/about/guidelines.xhtml

contribute to sample size calculations for an adequately powered trial, and additionally increase the chance of such a trial being successful. The study was not powered to detect differences between groups. Nevertheless, secondary outcomes included an exploration of between group differences in measures of symptom burden, antibiotic consumption and adverse events.[11]

Although not made explicit in the protocol, a comparison of the time to feeling fully recovered between groups was made, as pre-specified in the statistical analysis plan. This data was already being collected through the electronic diary, which had received ethical approval. A decision to conduct an exploratory analysis of the proportion of participants experiencing 'double sickening' (feeling fully recovered and then subsequently feeling unwell within 2 weeks of trial enrolment) was made after the statistical analysis plan had been completed, but before the trial data had been reviewed or analyses commenced. The concept of double sickening has been noted in acute respiratory illness. [14] A substantial amendment was approved by the ethics committee after trial recruitment had started to allow the trial team to interview recruiters to the trial, as part of the study's feasibility assessment.

Sample size and randomization

A sample size of 45 participants was chosen to enable estimation of a loss-to-follow-up rate of 20% with a confidence interval of 9.6 to 34.6%.[11] Sample sizes of between 24[15] and 50[16] have been suggested for pilot studies.

The randomisation sequence was generated using Stata v15.1 (StataCorp LP, College station, TX) by a statistician at the Nuffield Department of Primary Care Health Sciences, University of Oxford, using computer generated block randomisation with variable block size. The sequence was password-protected and accessible only by the statistician. Participants were



For peer review only - http://bmjopen.bmj.com/site/about/guidelines.xhtml

randomised by recruiting clinicians through the click of a button on the electronic trial registration page, which was created using Research Electronic Data Capture (REDCap).

Statistical methods

Unless otherwise stated, primary outcomes are presented as proportions and percentages for each trial group and for the entire trial population.

The number of antibiotic courses (or part thereof) consumed by participants in each group at 2 weeks and 4 weeks was compared using Poisson regression and reported using incidence rate ratios. The difference in the duration of symptoms rated moderately bad or worse between groups was compared using negative binomial regression; incidence rate ratios are reported and a Kaplan Meier graph plotted. The mean symptom burden scores (from day 2 to day 4 inclusive) were compared between groups using linear regression, and reported using mean differences. The difference in time to feeling fully recovered between groups was compared using the Cox proportional-hazards model; hazard ratios are presented and a Kaplan Meier graph was plotted. Each estimate was adjusted for study site.

A risk ratio and 95% confidence interval was used to estimate the difference in the proportion of participants consuming a course of antibiotics (or part thereof) between groups at two weeks and four weeks, the difference in the proportion of adverse events between groups at both two weeks and four weeks, and to compare the proportion of participants in each group experiencing double sickening. It was not possible to use log binomial regression, as originally planned, to compare the proportion of participants consuming antibiotics between groups as the model would not converge. A risk ratio and 95% confidence interval was used in lieu of Fisher's exact test (as originally planned) to estimate the difference in proportion of adverse events between groups in order to generate 95% confidence intervals rather than p



For peer review only - http://bmjopen.bmj.com/site/about/guidelines.xhtml

values. As this study was not powered to detect differences between groups, we had pre-specified that p values would not be reported.

Intention to treat analysis was conducted for all analyses and estimates are presented with their 95% confidence intervals. Due to the small sample size and the fact that this is a feasibility study, no subgroup or sensitivity analyses were conducted.

Patient and Public Involvement

Four PPI contributors have been closely involved with the study from the outset, two of whom sit on the Trial Steering Committee. During trial conception and development, they confirmed that the topic chosen was important. They critiqued all public facing trial documentation to ensure that it was free of jargon, and their feedback was incorporated. They were also involved in the design of the electronic diary, helping to ensure that it was user-friendly. PPI contributors unanimously felt that the diary would not be onerous for participants; and suggested it may make the experience of participating in the trial more meaningful, stating "…completing the symptom diary hooks people in. It makes them feel that they are contributing." The results of the study have been shared with PPI contributors, allowing them to give their views on ways to explain the findings, which have been incorporated into the discussion. PPI contributors are also helping to inform the dissemination of the study findings.

For peer review only



For peer review only - http://bmjopen.bmj.com/site/about/guidelines.xhtml

## Results

Baseline data

Baseline information was completed electronically by participants upon accessing the electronic diary on day one of enrolment, prior to inputting their day one symptom ratings. The mean age of participants was 48.3 years (range: 18 to 81 years). The mean age of women in the immediate cranberry and delayed antibiotics group was lower than the other two groups. Most women had experienced a previous UTI (85.4%, 35/41). 75.6% (31/41) of respondents were expecting antibiotics. 61% (25/41) believed that cranberry could help their symptoms, whilst 24.4% (10/41) believed that their symptoms could improve without antibiotics (table 1).

**Table 1** – Baseline Characteristics of Participants

| Proportion (%), unless otherwise specified | Group 1 (n=15) | Group 2 (n=15 ) | Group 3 (n=16) | Total (n=46 ) |
|---|---|---|---|---|
| **Age in years**, Mean (SD) [range] | 52.4 (21.4) [18-81] | 52.4 (22.4) [19-81] | 40.8 (14.0) [20-63] | 48.3 (19.9) [18-81] |
| **Previous UTI** | | | | |
| Yes | 12/15 (80%) | 12/13 (92.3%) | 11/13 (84.6%) | 35/41 (85.4%) |
| No | 3/15 (20%) | 1/13 (7.7%) | 2/13 (15.4%) | 6/41 (14.6) |
| Missing | 0/15 | 2/15 | 3/16 | 5/46 |
| **Previous UTI in the past year** | | | | |
| Yes | 7/15 (46.7%) | 6/13 (46.2%) | 6/13 (46.2%) | 19/41 (46.3%) |
| No | 5/15 (33.3%) | 6/13 (46.2%) | 5/13 (38.5%) | 16/41 (39.0%) |
| N/A *(Have never had a previous UTI)* | 3/15 (20%) | 1/13 (7.7%) | 2/13 (15.4%) | 6/41 (14.6%) |
| Missing | 0/15 | 2/15 | 3/16 | 5/46 |
| **If had a UTI in the past year, number of months since last UTI – mean (SD), median [range]** | 5.4 (4.5), 4 [0-12] n=7 | 5 (4.2), 5 [0-11] n=5 | 7 (3.6), 7 [3-12] n=5 | 5.8 (4.2), 5 [0-12] n=17 |
| Can't remember | 0/15 (0%) | 1/13 (7.7%) | 1/12 (8.3%) | 2/40 (5%) |
| Not applicable | 8/15 (53.3%) | 7/13 (53.9%) | 6/12 (50%) | 21/40 (52.5%) |



For peer review only - http://bmjopen.bmj.com/site/about/guidelines.xhtml

| | | | | |
|---|---|---|---|---|
| Missing | 0/15 | 2/15 | 4/16 | 6/46 |
| **Number of UTIs in the past year:** | | | | |
| Zero | 5/15 (33.3%) | 7/13 (53.9%) | 4/13 (30.8%) | 16/41 (39.0%) |
| One | 3/15 (20%) | 2/13 (15.4%) | 5/13 (38.5%) | 10/41 (24.4%) |
| Two | 2/15 (13.3%) | 2/13 (15.4%) | 1/13 (7.7%) | 5/41 (12.2%) |
| Three or more | 2/15 (13.3%) | 1/13 (7.7%) | 1/13 (7.7%) | 4/41 (9.8%) |
| Not applicable *(Have never had a previous UTI)* | 3/15 (20%) | 1/13 (7.7%) | 2/13 (15.4%) | 6/41 (14.6%) |
| Missing | 0/15 | 2/15 | 3/16 | 5/46 |
| **Were you expecting antibiotic treatment?** | | | | |
| Yes | 13/15 (86.7%) | 10/13 (76.9%) | 8/13 (61.5%) | 31/41 (75.6%) |
| No | 0/15 (0%) | 1/13 (7.7%) | 1/13 (7.7%) | 2/41 (4.9%) |
| Unsure | 2/15 (13.3%) | 2/13 (15.4%) | 4/13 (30.8%) | 8/41 (19.5%) |
| Missing | 0/15 | 2/15 | 3/16 | 5/46 |
| **Were you expecting tests/investigations?** | | | | |
| Yes | 12/15 (80%) | 10/13 (76.9%) | 8/13 (61.5%) | 30/41 (73.2%) |
| No | 3/15 (20%) | 3/13 (23.1%) | 4/13 (30.8%) | 10/41 (24.4%) |
| Unsure | 0/15 (0%) | 0/13 (0%) | 1/13 (7.7%) | 1/41 (2.4%) |
| Missing | 0/15 | 2/15 | 3/16 | 5/46 |
| **Were you expecting advice?** | | | | |
| Yes | 12/15 (80%) | 11/12 (91.7%) | 13/13 (100%) | 36/40 (90%) |
| No | 2/15 (13.3%) | 1/12 (8.3%) | 0/13 (0%) | 3/40 (7.5%) |
| Unsure | 1/15 (6.7%) | 0/12 (0%) | 0/13 (0%) | 1/40 (2.5%) |
| Missing | 0/15 | 3/15 | 3/16 | 6/46 |
| **Were you expecting something else?** | | | | |
| Yes | 0/15 (0%) | 1/13 (7.7%) - *Information and prescription (1 participant)* | 0/13 (0%) | 1/41 (2.4%) |
| No | 15/15 (100%) | 10/13 (76.9%) | 11/13 (84.6%) | 36/41 (87.8%) |



For peer review only - http://bmjopen.bmj.com/site/about/guidelines.xhtml

| | | | | |
|---|---|---|---|---|
| Unsure | 0/15 (0%) | 2/13 (15.4%) | 2/13 (15.4%) | 4/41 (9.8%) |
| Missing | 0/15 | 2/15 | 3/16 | 5/46 |
| **Was your last UTI treated with antibiotics?** | | | | |
| Yes | 8/15 (53.3%) | 9/13 (69.2%) | 10/13 (76.9%) | 27/41 (65.9%) |
| No | 4/15 (26.7%) | 3/13 (23.1%) | 1/13 (7.7%) | 8/41 (19.5%) |
| Not applicable | 3/15 (20%) | 1/13 (7.7%) | 2/13 (15.4%) | 6/41 (14.6%) |
| Missing | 0/15 | 2/15 | 3/16 | 5/46 |
| **Was your last UTI treated with something other than antibiotics?** | | | | |
| Yes | 2/15 (13.3%) - *Cystitis sachets (2 participants)* | 1/13 (7.7%) - *Cystitis sachets (1 participant)* | 0/13 (0%) | 3/41 (7.3%) |
| No | 10/15 (66.7%) | 11/13 (84.6%) | 11/13 (84.6%) | 32/41 (78.1%) |
| Not applicable | 3/15 (20%) | 1/13 (7.7%) | 2/13 (15.4%) | 6/41 (14.6%) |
| Missing | 0/15 | 2/15 | 3/16 | 5/46 |
| **Did you receive no treatment for your last UTI?** | | | | |
| Yes | 1/15 (6.7%) | 0/13 (0%) | 0/13 (0%) | 1/41 (2.4%) |
| No | 11/15 (73.3%) | 12/13 (92.3%) | 11/13 (84.6%) | 34/41 (82.9%) |
| Not applicable | 3/15 (20%) | 1/13 (7.7%) | 2/13 (15.4%) | 6/41 (14.6%) |
| Missing | 0/15 | 2/15 | 3/16 | 5/46 |
| **Unable to remember how last UTI was treated** | | | | |
| Yes | 1/15 (6.7%) | 1/13 (7.7%) | 1/13 (7.7%) | 3/41 (7.3%) |
| No | 11/15 (73.3%) | 11/13 (84.6%) | 10/13 (76.9%) | 32/41 (78.1%) |
| Not applicable | 3/15 (20%) | 1/13 (7.7%) | 2/13 (15.4%) | 6/41 (14.6%) |
| Missing | 0/15 | 2/15 | 3/16 | 5/46 |
| **Treatments tried before consulting primary care** | | | | |
| *Cranberry products* | | | | |
| Yes | 2/15 (13.3%) | 3/13 (23.1%) | 3/13 (23.1%) | 8/41 (19.5%) |
| No | 13/15 (86.7%) | 10/13 (76.9%) | 10/13 (76.9%) | 33/41 (80.5%) |
| Missing | 0/15 | 2/15 | 3/16 | 5/46 |



For peer review only - http://bmjopen.bmj.com/site/about/guidelines.xhtml

| | | | | |
|---|---|---|---|---|
| *Other fruit juice* | | | | |
| Yes | 1/15 (93.3%) | 1/13 (7.7%) | 0/13 (0%) | 2/41 (4.9%) |
| No | 14/15 (93.3%) | 12/13 (92.3%) | 13/13 (100%) | 39/41 (95.1%) |
| Missing | 0/15 | 2/15 | 3/16 | 5/46 |
| *Bicarbonate solution* | | | | |
| Yes | 0/15 (0%) | 0/13 (0%) | 0/13 (0%) | 0/41 (0%) |
| No | 15/15 (100%) | 13/13 (100%) | 13/13 (100%) | 41/41 (100%) |
| Missing | 0/15 | 2/15 | 3/16 | 5/46 |
| *Sodium/potassium citrate (cystitis sachets)* | | | | |
| Yes | 3/15 (20%) | 7/13 (53.9%) | 1/13 (7.7%) | 11/41 (26.8%) |
| No | 12/15 (80%) | 6/13 (46.2%) | 12/13 (92.3%) | 30/41 (73.2%) |
| Missing | 0/15 | 2/15 | 3/16 | 5/46 |
| *Uvacin/Uva ursi (bearberry)* | | | | |
| Yes | 0/15 (0%) | 0/13 (0%) | 0/13 (0%) | 0/41 (0%) |
| No | 15/15 (100%) | 13/13 (100%) | 13/13 (100%) | 41/41 (100%) |
| Missing | 0/15 | 2/15 | 3/16 | 5/46 |
| *Pain relief (e.g. paracetamol/Ibuprofen)* | | | | |
| Yes | 6/15 (40%) | 7/13 (53.9%) | 4/13 (30.8%) | 17/41 (41.5%) |
| No | 9/15 (60%) | 6/13 (46.2%) | 9/13 (69.2%) | 24/41 (58.5%) |
| Missing | 0/15 | 2/15 | 3/16 | 5/46 |
| *Other treatment used?* | | | | |
| Yes | 2/15 (13.3%) – *Water (2 participants)* | 2/13 (15.4%) – *Tea tree in bath (1 participant) Sachets (1 participant)* | 2/13 (15.4%) – *Water (2 participants)* | 6/41 (14.6%) |
| No | 13/15 (86.7%) | 11/13 (84.6%) | 11/13 (84.6%) | 35/41 (85.4%) |
| Missing | 0/15 | 2/15 | 3/16 | 5/46 |
| *None* | | | | |
| Yes | 7/15 (46.7%) | 2/13 (15.4%) | 5/13 (38.5%) | 14/41 (34.2%) |
| No | 8/15 (53.3%) | 11/13 (84.6%) | 8/13 (61.5%) | 27/41 (65.9%) |
| Missing | 0/15 | 2/15 | 3/16 | 5/46 |
| **Number of days symptomatic** | | | | |



For peer review only - http://bmjopen.bmj.com/site/about/guidelines.xhtml

| | | | | |
|---|---|---|---|---|
| 0 days | 1/15 (6.7%) | 2/13 (15.4%) | 1/13 (7.7%) | 4/41 (9.8%) |
| 1 day | 3/15 (20%) | 1/13 (7.7%) | 2/13 (15.4%) | 6/41 (14.6%) |
| 2 days | 4/15 (26.7%) | 3/13 (23.1%) | 5/13 (38.5%) | 12/41 (29.3%) |
| 3 days | 2/15 (13.3%) | 2/13 (15.4%) | 4/13 (30.8%) | 8/41 (19.5%) |
| >3 days | 5/15 (33.3%) | 5/13 (38.5%) | 1/13 (7.7%) | 11/41 (26.8%) |
| Missing | 0/15 | 2/15 | 3/16 | 5/46 |
| **Belief that cranberry can help symptoms** | | | | |
| Yes | 10/15 (66.7%) | 7/13 (53.9%) | 8/13 (61.5%) | 25/41 (61%) |
| No | 1/15 (6.7%) | 0/13 (0%) | 0/13 (0%) | 1/41 (2.4%) |
| Unsure | 4/15 (26.7%) | 6/13 (46.2%) | 5/13 (38.5%) | 15/41 (36.6%) |
| Missing | 0/15 | 2/15 | 3/16 | 5/46 |
| **Belief that symptoms can get better without antibiotics** | | | | |
| Yes | 5/15 (33.3%) | 1/13 (7.7%) | 4/13 (30.8%) | 10/41 (24.4%) |
| No | 4/15 (26.7%) | 4/13 (30.8%) | 2/13 (15.4%) | 10/41 (24.4%) |
| Unsure | 6/15 (40%) | 8/13 (61.5%) | 7/13 (53.9%) | 21/41 (51.2%) |
| Missing | 0/15 | 2/15 | 3/16 | 5/46 |

Group 1 – Immediate antibiotics alone (control); Group 2 – Immediate antibiotics and immediate cranberry; Group 3 – Immediate cranberry and delayed antibiotics.

For peer review only

## Primary Outcomes

Forty-six women were recruited to the trial, surpassing the target of 45 (See figure 1). Of the 21 women who were eligible but declined to take part, the most common reason for declining was not wishing to be randomised to receive delayed antibiotics (42.9%, 9/21). The first participant was recruited on 11th July 2019 and the final participant was recruited on 2nd December 2019, with the final follow-up taking place on 30th December 2019. The findings



For peer review only - http://bmjopen.bmj.com/site/about/guidelines.xhtml

of qualitative interviews with 14 trial participants and eight trial recruiters will be presented elsewhere.

89.1% (41/46) of electronic diaries had some data entered by participants, whilst 69.6% (32/46) of electronic diaries were fully complete (table 2). There were no electronic diary failures necessitating the issue of paper diaries. A mean of 9.2 participants were recruited per month, which exceeded predicted recruitment of between four and eight participants each month. All GP practices that were invited to recruit to the trial agreed to participate (100%, 5/5), however, one GP practice withdrew prior to site opening as they predicted that they would no longer have capacity to support the trial.

**Table 2** – Primary Outcomes

| Proportion (%), unless otherwise specified | Group 1 (n=15) | Group 2 (n=15 ) | Group 3 (n=16) | Total (n=46 ) |
|---|---|---|---|---|
| **Number of diaries with some data points entered by participant by end of participant follow-up** | 15 (100%) | 13 (86.7%) | 13 (81.3%) | 41 (89.1%) |
| **Number of diaries fully completed by participant by end of participant follow-up** | 13 (86.7%) | 10 (66.7%) | 9 (56.3%) | 32 (69.6%) |
| **Paper diaries issued due to failure of electronic diary** | 0 (0%) | 0 (0%) | 0 (0%) | 0 (0%) |
| **Number randomised as a proportion of the target recruitment number** | 46/45 (102.2%) | | | |
| **Number randomised as a proportion of invited participants** | 46/120 (38.3%) | | | |
| **Number randomised as a proportion of eligible participants** | 46/67 (68.7%) | | | |
| No. patients invited | | | | 120 |
| No. patients eligible | | | | 67 |
| No. participants enrolled | 15 | 15 | 16 | 46 |
| No. participants randomised | 15 | 15 | 16 | 46 |
| No. recruited per month (n) | | | | 9.2 |
| **Number of participants lost to follow-up** | 0 (0%) | 2 (13.3%) | 1 (6.3%) | 3 (6.5%) |
| **Number of participants withdrawing from study** | 0 (0%) | 0 (0%) | 2 (12.5%) | 2 (4.4%) |
| **Side effects or problems related to cranberry consumption** | | | | |

For peer review only



For peer review only - http://bmjopen.bmj.com/site/about/guidelines.xhtml

| | | | | |
|---|---|---|---|---|
| Yes | 0/13 (0%) | 0/10 (0%) | 1/11 (9.1%) | 1/34 (2.9%) |
| No | 1/13 (7.7%) | 10/10 (100%) | 10/11 (90.9%) | 21/34 (61.8%) |
| Not applicable | 12/13 (92.3%) | 0/10 (0%) | 0/11 (0%) | 12/34 (35.3%) |
| Missing | 2/15 | 5/15 | 5/16 | 12/46 |
| **Problems completing the electronic diary** | | | | |
| No problem | 14/14 (100%) | 9/10 (90%) | 9/11 (81.8%) | 32/35 (91.4%) |
| Some problems | 0/14 (0%) | 1/10 (10%) | 2/11 (18.2%) | 3/35 (8.6%) |
| Lots of problems | 0/14 (0%) | 0/10 (0%) | 0/11 (0%) | 0/35 (0%) |
| Missing | 1/15 | 5/15 | 5/16 | 11/46 |
| **Problems using the scale of 0-50** | | | | |
| No problem | 12/14 (85.7%) | 9/9 (100%) | 10/12 (83.3%) | 31/35 (88.6%) |
| Some problems | 2/14 (14.3%) | 0/9 (0%) | 2/12 (16.7%) | 4/35 (11.4%) |
| Lots of problems | 0/14 (0%) | 0/9 (0%) | 0/12 (0%) | 0/35 (0%) |
| Missing | 1/15 | 6/15 | 4/16 | 11/46 |
| **Proportion of invited GP practices agreeing to participate in the trial** | 5/5 (100%) | | | |
| **Proportion of invited GP practices opened and recruiting to the trial** | 4/5 (80%) | | | |
| **Acceptability of the study to participants and recruiters, participants' experience of having a UTI and thoughts on self-help for UTIs.** | Interviews with trial participants and recruiters will be reported elsewhere. | | | |

Group 1 – Immediate antibiotics alone (control); Group 2 – Immediate antibiotics and immediate cranberry; Group 3 – Immediate cranberry and delayed antibiotics.

Only one participant reported a side effect or problem related to consuming cranberry capsules in the electronic diary (table 2). Few participants reported experiencing problems completing the electronic diary (8.6%, 3/35) or rating their symptoms using the scale of 0 to 50 (11.4%, 4/35).



For peer review only - http://bmjopen.bmj.com/site/about/guidelines.xhtml

Three participants were lost to follow-up (Figure 1). Loss to follow-up was determined to have occurred when a participant did not make any electronic diary entries and it was not possible to contact them at the 2 week follow-up call.

Two participants withdrew from the study. One participant was consented and randomly assigned to immediate cranberry capsules with delayed antibiotics. As soon as she received this assignment, she decided to withdraw from the study and did not receive the cranberry capsules. Another participant was consented and also randomised to immediate cranberry and delayed antibiotics. The day after being enrolled, she emailed the trial team asking to be removed from the mailing list (links to the electronic diary were sent by email). In light of this, the chief investigator withdrew the participant from the study.

Participants were able to provide free-text comments on the final day (day 14) of diary completion. Free-text boxes allowed women to provide general comments about the trial, as well as comments specifically about difficulties using the electronic diary, difficulties using the rating scale and any problems related to consuming cranberry capsules. Fourteen women provided comments.

Some women provided general feedback, including being "happy to help" with the study and finding the diary "very easy to complete." Two participants described some difficulty keeping up with filling in the diary *"Just time and keeping up with it that is all. Happy to have taken part." (*41-8, Immediate cranberry and delayed antibiotics). Another participant mentioned that she did not receive a link to complete the final set of diary questions, and one woman was unclear about the guidance regarding the need to continue completing the diary once symptoms had resolved for two consecutive days. Two women commented that the scale of 0 to 50 was "too wide a range."



For peer review only - http://bmjopen.bmj.com/site/about/guidelines.xhtml

One woman stated that she was sceptical about cranberry at first, but found that it "helped a lot" (32-11, Immediate cranberry and delayed antibiotics). One participant found that the combination of antibiotic with cranberry cleared her infection "quite quickly" (32-14, Immediate antibiotics and immediate cranberry), whilst another found that the antibiotic/cranberry combination resulted in her taking a shorter course of antibiotics than usual – *"Nice to only take three days of antibiotics not a week"* (23-3, Immediate antibiotics and immediate cranberry). One lady stated that despite starting with cranberry, she had to take antibiotics on day 2 as she was in pain (41-2, Immediate cranberry and delayed antibiotics).

Three adverse events were picked up from free-text comments (see Harms and Table 5 below).

**Secondary Outcomes**

Approximately one third of respondents reported contacting a healthcare practitioner within two weeks of being enrolled (36.4%, 12/33). Most of these women contacted their GP practice (GP or nurse at their surgery) - one woman reported liaising with a pharmacist, and another contacted an out of hours GP. No participants reported requiring Accident and Emergency or secondary care input. Just over half (54.3%, 25/46) of the urine samples were positive (organism growth greater than $10^5$/CFU).

Exploratory analyses of effectiveness were conducted, whilst recognising that the study was not powered for these analyses (table 3). For this reason, p values have not been reported and any differences detected between groups are inconclusive.



For peer review only - http://bmjopen.bmj.com/site/about/guidelines.xhtml

**Table 3** – Exploratory effectiveness outcomes

| | Proportion (%) of participants consuming a course of antibiotics (or part thereof) at 2 weeks [risk ratio (95% CI)] | Proportion (%) of participants consuming a course of antibiotics (or part thereof) at 4 weeks [risk ratio (95% CI)] | Mean (SD) number of courses of antibiotics consumed at 2 weeks [adjusted incidence rate ratio (95% CI)] † | Mean (SD) number of courses of antibiotics consumed at 4 weeks [adjusted incidence rate ratio (95% CI)] † | Median (IQR) number of days of symptoms rated moderately bad or worse [adjusted incidence rate ratio (95% CI)] † | Mean (SD) symptom burden from day 2 to 4 inclusive [adjusted mean difference (95% CI)] †¥ | Median (IQR) days to feeling fully recovered [adjusted hazard ratio (95% CI)] † | Proportion (%) of participants experiencing double sickening up to 2 weeks [risk ratio (95% CI)] |
|---|---|---|---|---|---|---|---|---|
| **Group 1** (Control) n=15 | 15/15 (100%)<br><br>Missing: 0/15 | 15/15 (100%)<br><br>Missing: 0/15 | 1.5 (0.5)<br><br>Missing: 0/15 | 1.6 (0.6)<br><br>Missing: 0/15 | 2 (1 to 2)<br><br>Missing: 0/15 | 6.3 (3.4)<br><br>Missing: 1/15 | 5 (4 to 6)<br><br>Missing: 0/15 | 6/14 (42.9%)<br><br>Missing: 1/15 |
| **Group 2** n=15 | 13/13 (100%)<br>[1 (1 to 1)]<br><br>Missing: 2/15 | 13/13 (100%)<br>[1 (1 to 1)]<br><br>Missing: 2/15 | 1.1 (0.3)<br>[0.7 (0.4 to 1.4 )]<br><br>Missing: 2/15 | 1.2 (0.4)<br>[0.7 (0.4 to 1.4)]<br><br>Missing: 2/15 | 2 (1 to 3)<br>[1.2 (0.7 to 2.1)]<br><br>Missing: 2/15 | 6.2 (8.0)<br>[0.6 (-4.0 to 5.3)]<br><br>Missing: 4/15 | 4 (3 to 6)<br>[1.7 (0.7 to 4.1)]<br><br>Missing: 2/15 | 3/11 (27.3%)<br>[0.6 (0.2 to 2.0)]<br><br>Missing: 4/15 |
| **Group 3** n=16 | 11/14 (78.6%)<br>[0.8 (0.6 to 1.0]<br><br>Missing: 2/16 | 11/14 (78.6%)<br>[0.8 (0.6 to 1.0]<br><br>Missing: 2/16 | 0.9 (0.6)<br>[0.6 (0.3 to 1.2 )]<br><br>Missing: 2/16 | 0.9 (0.6)<br>[0.6 (0.3 to 1.2)]<br><br>Missing: 2/16 | 4 (2 to 8)<br>[2.9 (1.7 to 5.1)]<br><br>Missing: 4/16 | 11.2 (8.5)<br>[7.9 (2.6 to 13.2)]<br><br>Missing: 7/16 | 13 (5 to 9)<br>[0.6 (0.2 to 1.4)]<br><br>Missing: 3/16 | 0/12 (0%)<br>[0 (N/A)]<br><br>Missing: 4/16 |

Group 1 – Immediate antibiotics alone (control); Group 2 – Immediate antibiotics and immediate cranberry; Group 3 – Immediate cranberry and delayed antibiotics. Group 2 and group 3 were compared with Group 1 (controls).

†Model adjusted for site

¥ The mean symptom burden for each participant was calculated by summing the total ratings for all symptoms between day 2 and day 4 (inclusive) and dividing this by the number of symptom ratings made by the participant between day 2 and day 4 (inclusive).

**Abbreviations:** SD: Standard Deviation; %: Percentage



For peer review only - http://bmjopen.bmj.com/site/about/guidelines.xhtml

There was a 20% reduction in the risk of consuming a course of antibiotics (or part thereof) in the immediate cranberry group (group 3), compared with the immediate antibiotics alone group (group 1- controls). This was the case at both 2 weeks and 4 weeks (risk ratio 0.8; 95% CI: 0.6 to 1.0). The number of courses of antibiotics consumed at 2 weeks and at 4 weeks was reduced in groups 2 (immediate antibiotics and immediate cranberry) and 3 (immediate cranberry and delayed antibiotics), compared with the control group (adjusted incidence rate ratios: 0.7; 95% CI: 0.4 to 1.4 and 0.6; 95% CI: 0.3 to 1.2 for group 2 and group 3, respectively). Participants in groups 2 and 3 had a longer duration of symptoms rated moderately bad or worse, compared with those in the control group (adjusted incidence rate ratios: 1.2; 95% CI: 0.7 to 2.1 and 2.9; 95% CI 1.7 to 5.1 for group 2 and group 3, respectively). The mean symptom burden (from day 2 to day 4 inclusive) was reduced in group 2 participants (adjusted mean difference: 0.6; 95% CI: -4.0 to 5.3) and increased in group 3 participants (adjusted mean difference: 7.9; 95% CI 2.6 to 13.2), compared with the control group. Similarly, the time to feeling recovered was reduced in group 2 (adjusted hazard ratio 1.7; 95% CI: 0.7 to 4.1) but increased in group 3 (adjusted hazard ratio 0.6; 95% CI: 0.2 to 1.4), compared with controls. Of women randomly assigned to take antibiotics alone (controls), one third of respondents reported consuming cranberry products (33.3%, 4/12) (table 4).

For peer review only

**Table 4** – Secondary outcomes

| Proportion (%), unless otherwise specified | Group 1 (n=15) | Group 2 (n=15 ) | Group 3 (n=16) | Total (n=46 ) |
|---|---|---|---|---|
| **Any of the following treatments used in the past 2 weeks (not including study medication)** | | | | |
| *Cranberry products* | | | | |
| Yes | 4/12 (33.3%) | 2/9 (22.2%) | 2/9 (22.2%) | 8/30 (26.7%) |
| No | 8/12 (66.7%) | 7/9 (77.8%) | 7/9 (77.8%) | 22/30 (73.3%) |
| Missing | 3/15 | 6/15 | 7/16 | 16/46 |
| *Other fruit juice* | | | | |



For peer review only - http://bmjopen.bmj.com/site/about/guidelines.xhtml

| | | | | |
|---|---|---|---|---|
| Yes | 0/12 (0%) | 0/9 (0%) | 0/9 (0%) | 0/30 (0%) |
| No | 12/12 (100%) | 9/9 (100%) | 9/9 (100%) | 30/30 (100%) |
| Missing | 3/15 | 6/15 | 7/16 | 16/46 |
| *Bicarbonate solution* | | | | |
| Yes | 0/12 (0%) | 0/9 (0%) | 0/9 (0%) | 0/30 (0%) |
| No | 12/12 (100%) | 9/9 (100%) | 9/9 (100%) | 30/30 (100%) |
| Missing | 3/15 | 6/15 | 7/16 | 16/46 |
| *Sodium/potassium citrate (cystitis sachets)* | | | | |
| Yes | 0/12 (0%) | 0/9 (0%) | 1/9 (11.1%) | 1/30 (3.3%) |
| No | 12/12 (100%) | 9/9 (100%) | 8/9 (88.9%) | 29/30 (96.7%) |
| Missing | 3/15 | 6/15 | 7/16 | 16/46 |
| *Uvacin/Uva ursi (bearberry)* | | | | |
| Yes | 0/12 (0%) | 0/9 (0%) | 0/9 (0%) | 0/30 (0%) |
| No | 12/12 (100%) | 9/9 (100%) | 9/9 (100%) | 30/30 (100%) |
| Missing | 3/15 | 6/15 | 7/16 | 16/46 |
| *Pain relief (e.g. paracetamol/Ibuprofen)* | | | | |
| Yes | 5/12 (33.3%) | 1/9 (11.1%) | 5/9 (55.6%) | 11/30 (36.7%) |
| No | 7/12 (58.3%) | 8/9 (88.9%) | 4/9 (44.4%) | 19/30 (63.3%) |
| Missing | 3/15 | 6/15 | 7/16 | 16/46 |
| *Other* | | | | |
| Yes | 0/12 (0%) | 0/9 (0%) | 0/9 (0%) | 0/30 (0%) |
| No | 12/12 (100%) | 9/9 (100%) | 9/9 (100%) | 30/30 (100%) |
| Missing | 3/15 | 6/15 | 7/16 | 16/46 |
| *None* | | | | |
| Yes | 5/12 (41.7%) | 6/9 (66.7%) | 3/9 (33.3%) | 14/30 (46.7%) |
| No | 7/12 (58.3%) | 3/9 (33.3%) | 6/9 (66.7%) | 16/30 (53.3%) |
| Missing | 3/15 | 6/15 | 7/16 | 16/46 |
| **Time off paid work or normal activities** | | | | |
| Yes | 5/13 (38.5%) | 2/10 (20%) | 3/9 (33.3%) | 10/32 (31.3%) |
| No. of days, median (IQR) | 1 | 5 | 2 | 2 |



For peer review only - http://bmjopen.bmj.com/site/about/guidelines.xhtml

| | | | | |
|---|---|---|---|---|
| | (1-2) | (2-8) | (1-2) | (1-2) |
| No | 8/13 (61.5%) | 8/10 (80%) | 6/9 (66.7%) | 22/32 (68.8%) |
| Missing | 2/15 | 5/15 | 7/16 | 14/46 |
| **Healthcare professional consulted at GP practice** | | | | |
| Yes | 6/13 (46.2%) | 1/8 (12.5%) | 5/12 (41.7%) | 12/33 (36.4%) |
| No | 7/13 (53.9%) | 7/8 (87.5%) | 7/12 (58.3%) | 21/33 (63.6%) |
| Missing | 2/15 | 7/15 | 4/16 | 13/46 |
| *GP at surgery* | | | | |
| Yes | 3/13 (23.1%) | 0/8 (0%) | 2/12 (16.7%) | 5/33 (15.2%) |
| No. of times, median (IQR) | 1 (1-1) | N/A | 1 (1-1) | 1 (1-1) |
| No | 3/13 (23.1%) | 1/8 (12.5%) | 3/12 (25%) | 7/33 (21.2%) |
| Not applicable | 7/13 (53.9%) | 7/8 (87.5%) | 7/12 (58.3%) | 21/33 (63.6%) |
| Missing | 2/15 | 7/15 | 4/16 | 13/46 |
| *Nurse at surgery* | | | | |
| Yes | 2/13 (15.4%) | 1/8 (12.5%) | 1/12 (8.3%) | 4/33 (12.1%) |
| No. of times, median (IQR) | 1 (1-1) | 2 (2-2) | 1 (1-1) | 1 (1-1.5) |
| No | 4/13 (30.8%) | 0/8 (0%) | 4/12 (33.3%) | 8/33 (24.2%) |
| Not applicable | 7/13 (53.9%) | 7/8 (87.5%) | 7/12 (58.3%) | 21/33 (63.6%) |
| Missing | 2/15 | 7/15 | 4/16 | 13/46 |
| *GP at home* | | | | |
| Yes | 0/13 (0%) | 0/8 (0%) | 0/12 (0%) | 0/33 (0%) |
| No. of times, median (IQR) | N/A | N/A | N/A | N/A |
| No | 6/13 (46.2%) | 1/8 (12.5%) | 5/12 (41.7%) | 12/33 (36.4%) |
| Not applicable | 7/13 (53.9%) | 7/8 (87.5%) | 7/12 (58.3%) | 21/33 (63.6%) |
| Missing | 2/15 | 7/15 | 4/16 | 13/46 |
| *OOH doctor* | | | | |
| Yes | 1/13 (7.7%) | 0/8 (0%) | 0/12 (0%) | 1/33 (3.0%) |
| No. of times, median (IQR) | 1 (1-1) | N/A | N/A | 1 (1-1) |
| No | 5/13 (38.5%) | 1/8 (12.5%) | 5/12 (41.7%) | 11/33 (33.3%) |
| Not applicable | 7/13 (53.9%) | 7/18 (87.5%) | 7/12 (58.3%) | 21/33 (63.6%) |



For peer review only - http://bmjopen.bmj.com/site/about/guidelines.xhtml

For peer review only

| | | | | |
|---|---|---|---|---|
| Missing | 2/15 | 7/15 | 4/16 | 13/46 |
| *Other* | | | | |
| Yes | 0/13 (0%) | 0/8 (0%) | 2/12 (16.7%) – *Pharmacist (1 participant), Phoned GP (1 participant)* | 2/33 (6.1%) |
| No. of times, median (IQR) | N/A | N/A | 1 (1-1) | 1 (1-1) |
| No | 6/13 (46.2%) | 1/8 (12.5%) | 3/12 (25%) | 10/33 (30.3%) |
| Not applicable | 7/13 (53.9%) | 7/8 (87.5%) | 7/12 (58.3%) | 21/33 (63.6%) |
| Missing | 2/15 | 7/15 | 4/16 | 13/46 |
| **Healthcare professional consulted at A&E** | | | | |
| Yes | 0/13 (0%) | 0/10 (0%) | 0/12 (0%) | 0/35 (0%) |
| No | 13/13 (100%) | 10/10 (100%) | 12/12 (100%) | 35/35 (100%) |
| Missing | 2/15 | 5/15 | 4/16 | 11/46 |
| No. of times, median (IQR) | N/A | N/A | N/A | N/A |
| **Seen by a specialist (excluding hospital admission)** | | | | |
| Yes | 0/13 (0%) | 0/10 (0%) | 0/12 (0%) | 0/35 (0%) |
| No | 13/13 (100%) | 10/10 (100%) | 12/12 (100%) | 35/35 (100%) |
| Missing | 2/15 | 5/15 | 4/16 | 11/46 |
| No. of times, median (IQR) | N/A | N/A | N/A | N/A |
| **Admitted to hospital** | | | | |
| Yes | 0/13 (0%) | 0/10 (0%) | 0/12 (0%) | 0/35 (0%) |
| No | 13/13 (100%) | 10/10 (100%) | 12/12 (100%) | 35/35 (100%) |
| Missing | 2/15 | 5/15 | 4/16 | 11/46 |
| No. of nights, median (IQR) | N/A | N/A | N/A | N/A |
| **Positive mid-stream urine culture** | | | | |
| Yes | 10/15 (66.7%) | 7/15 (46.7%) | 8/16 (50%) | 25/46 (54.3%) |
| No | 5/15 (33.3%) | 8/15 (53.3%) | 8/16 (50%) | 21/46 (45.7%) |
| Missing | 0/15 | 0/15 | 0/16 | 0/46 |

For peer review only



For peer review only - http://bmjopen.bmj.com/site/about/guidelines.xhtml

| | |
|---|---|
| **Proportion with a positive mid-stream urine culture** | 54.3% |

All outcomes reported in relation to the two weeks following trial enrolment.

Group 1 – Immediate antibiotics alone (control); Group 2 – Immediate antibiotics and immediate cranberry; Group 3 – Immediate cranberry and delayed antibiotics.

An exploratory analysis was conducted to compare the proportion of participants experiencing 'double sickening' between groups at two weeks. Double sickening was defined as a participant selecting the relevant checkbox in the electronic diary to confirm that they felt fully recovered, then subsequently indicating through checkbox responses that they no longer felt fully recovered. Given the short period of time over which this was assessed (2 weeks from enrolment), it is likely that these double sickening episodes represent incompletely treated infection, rather than a new infection. Compared with the control group, group 2 participants had a reduced risk of experiencing double sickening (risk ratio 0.6; 95% CI: 0.2 to 2.0), whilst none of the group 3 participants experienced double sickening.

<u>Harms</u>

Seven adverse events occurred (table 5). Adverse events four and five occurred in the same participant. Two adverse event descriptions sounded like *possible* pyelonephritis (numbers three and four). However, one of these adverse events occurred in a participant whose MSU was negative, who reported feeling fully recovered on day 5 of the study and who did not contact a healthcare practitioner up to 28 days after enrolment. The second occurred in a participant who subsequently contacted her GP practice, however, her clinical syndrome was not reported as pyelonephritis by the consulting healthcare practitioner and the participant's symptoms settled with increased fluid intake alone. There were no serious adverse events.



For peer review only - http://bmjopen.bmj.com/site/about/guidelines.xhtml

**Table 5** – List of Adverse Events

| Adverse Event Number | Group | Adverse Event | Outcome | Severity | Relationship to study product | Comments |
|---|---|---|---|---|---|---|
| 1 | Immediate cranberry and delayed antibiotics | Felt sick | Resolved | Mild | Possibly related | Participant mentioned that she was unsure whether the sickness symptom was related to cranberry or her UTI. |
| 2 | Immediate antibiotics alone | Reaction after taking one dose of antibiotic | Resolved | Uncertain | Not related | Not applicable |
| 3 | Immediate cranberry and delayed antibiotics | General feeling of being unwell, terrible back ache and stomach ache at times. Headache and nausea | Resolved | Moderate | Possibly related | Possible pyelonephritis, however, no growth on MSU and the participant did not contact a healthcare practitioner up to 28 days. |
| 4 | Immediate antibiotics and immediate cranberry | Suprapubic pain, dysuria, backache, cloudy urine and single vomit | Resolved | Moderate | Possibly related | Possible pyelonephritis from description of symptoms, although not reported as such by healthcare practitioner. MSU positive - E.coli $10^5$ CFU/ml. |
| 5 | Immediate antibiotics and immediate cranberry | Vomiting after second course of Nitrofurantoin started | Resolved | Mild | Possibly related | Vomit after second course of antibiotics started. Antibiotics stopped and participant encouraged to push fluids. Symptoms settled without further antibiotics being prescribed. |
| 6 | Immediate cranberry and delayed antibiotics | Feeling unwell, ache in lower back | Resolved | Moderate | Possibly related | Symptoms unlikely to be related to the study product, as the participant was recruited in the morning and the symptoms started the same day in the afternoon. Participant reported feeling fully recovered on day 2 and did not contact healthcare practitioner up to 28 days. MSU positive > E.coli $10^5$ CFU/ml |
| 7 | Immediate antibiotics alone | Tummy upset that started 3 days after starting Ciprofloxacin (second course of antibiotics) | Resolved | Mild | Not related | Not applicable |

**Abbreviations:** MSU: Mid-stream urine; CFU: Colony forming unit

The difference in the proportion of adverse events between groups was compared, although the feasibility trial was not powered to detect differences between groups. At two weeks, the point estimates indicated an increased risk of experiencing an adverse event for participants in group 2 (risk ratio 1.2; 95% CI: 0.1 to 16.7) and group 3 (risk ratio 3.5; 95% CI: 0.4 to 29.4) compared with the control group, although the 95% confidence intervals were wide. At four weeks, the risk of experiencing an adverse event in group 2 was lower than for the control group (risk ratio 0.6; 95% CI: 0.1 to 5.7). The risk remained higher for participants in group 3 compared with controls at four weeks (risk ratio 1.7; 95% CI: 0.3 to 8.8) (supplementary file).

For peer review only



For peer review only - http://bmjopen.bmj.com/site/about/guidelines.xhtml

## Discussion

In this open-label, feasibility randomised trial, we recruited beyond target and at a rate faster than predicted. Overall, participants found the trial procedures and interventions acceptable; few reported problems consuming cranberry capsules or using the electronic symptom diary.

<u>Strengths and Weaknesses</u>

To our knowledge, this is the first trial to use cranberry capsules alone and combined with antibiotics as an *acute* treatment for UTI. The trial processes were designed, in discussion with PPI contributors, to be minimally burdensome for participants and recruiters, which may have contributed to the faster than anticipated recruitment, and to recruiting beyond target.

Using a participant-completed diary led to missing data, and in particular, incomplete baseline data. We also collected very limited demographic data. Missing data means that we do not have a full picture of all of the participants' experiences and outcomes. However, insights gained from the trial combined with the qualitative interviews (to be reported elsewhere) should help to optimise future trial design and minimise missing data in a future study.

Some may describe the open-label design as a weakness. However, this was designed to be a pragmatic trial with procedures closely aligned with real-world practice. We were therefore not prescriptive about the antibiotic issued, nor the duration, recognising that clinicians' prescribing is influenced by a host of factors, including a patient's past medical history and previous UTI history. Despite this, we appreciate that there is a lack of standardisation of antibiotic prescribing between groups. Furthermore, participants who missed diary entries

For peer review only

were able to complete the diary retrospectively; retrospective entries are likely to be less accurate than real-time diary completion.

We did not control participants' consumption of other sources of PAC with Type A linkages, namely blueberry. However, unless participants regularly consumed large amounts of blueberry it is unlikely that this would have had a significant impact on the results. Interestingly, despite advice not to, one third of women in the immediate antibiotics alone group reported consuming cranberry products during the trial. This highlights the challenge of trying to control external influences in a trial, particularly when this is in conflict with a participant's usual behaviour.

<u>Comparison with the existing literature</u>

No study participants developed pyelonephritis. A number of trials of non-antibiotic treatments have reported higher levels of pyelonephritis in participants not assigned to an antibiotic arm.[3 5-7] The absence of cases of pyelonephritis in this trial may well have been due to the small sample size, but may also have resulted from all participants receiving a prescription for antibiotics, whether immediate or delayed antibiotics. In a trial of 382 women with uncomplicated UTIs evaluating the use of ibuprofen and the herbal extract Uva ursi, all participants received delayed antibiotics and there were no cases of pyelonephritis[4]. The proportion of participants lost to follow-up or withdrawing from this feasibility trial (11%) is roughly comparable with analogous trials, which report between 1.3%[17] and 16%[7]. Of note, loss to follow-up is defined differently in different studies. The proportion of participants with a positive urine culture (54.3%) was lower than the proportion reported in some analogous trials (range of 32%[4] to 80%[17]). Of note, this trial used a cut-off threshold for urine culture positivity of $10^5$ CFU/ml, whilst most other trials were aligned with European guidance and used a threshold of greater than $10^2$ or $10^3$CFU/ml.



For peer review only - http://bmjopen.bmj.com/site/about/guidelines.xhtml

Whilst not powered to detect differences between groups, in line with the findings of similar trials, participants in this feasibility trial not receiving immediate antibiotics (group 3) consumed fewer antibiotics but had a higher symptom burden. However, participants in group 3 were less likely to experience double sickening. Interestingly, Gagyor and colleagues[6] found that significantly fewer participants receiving ibuprofen compared with antibiotics experienced UTI recurrence between days 15 to 28 [percentage mean difference -5.3% (95%CI: -10.2% to -0.4%), p = 0.049]. However, the difference between groups did not reach statistical significance when all recurrent UTIs from day 1 to day 28 were assessed [percentage mean difference -2.8% (95%CI: -8.7% to 3.1%), p = 0.41].

A Cochrane review in 2012 found that overall, treatment with cranberry did not significantly reduce *recurrent* UTIs (n=13 studies; risk ratio 0.86; 95% CI: 0.71 to 1.04).[18] This was also the case in a sub-group analysis that focused on women with UTI (n=4 studies; risk ratio 0.74; 95% CI: 0.42 to 1.31). However, the review authors acknowledged that many included studies had high rates of participant withdrawal/loss to follow-up of up to 55%, largely attributed to difficulty adhering to drinking large volumes of cranberry juice on a daily basis. Furthermore, there was a lack of standardisation and poor reporting of the amount of cranberry extract - and specifically of proanthocyanidin (PAC) - in the interventions used. In this feasibility trial, we used cranberry capsules with a standardised amount of PAC, for short-term use in the context of acute UTI. These key differences may have contributed to better adherence of participants to the study intervention.

Implications for clinicians, researchers and policymakers



For peer review only - http://bmjopen.bmj.com/site/about/guidelines.xhtml

452 This feasibility trial was not powered to detect differences between groups, thus, no definite
453 inferences about the effectiveness or safety of cranberry in the context of acute
454 uncomplicated UTI can be made. If cranberry extract is found to be safe and effective in the
455 management of acute UTIs through robust and adequately powered trials, it could represent
456 an important health intervention for this common condition and has the potential to
457 significantly reduce antibiotic consumption.
458 There is a need for adequately powered, randomised clinical trials to provide definitive
459 evidence of whether cranberry extract is effective in reducing antibiotic use and managing
460 symptoms of acute UTI. Whilst this feasibility trial does not provide evidence of the
461 effectiveness or safety of cranberry extract in the context of acute UTI, the exploratory
462 analyses suggest that it would be worthwhile to explore these as yet unanswered questions
463 through an adequately powered trial. Such trials should use cranberry products with
464 standardised amounts of PAC. Participants should be willing to stop all non-trial cranberry
465 products for the duration of the study; as a minimum, the use of non-trial cranberry products
466 by participants should be captured.
467

468 Conclusion

469 It is feasible to conduct a randomised trial of similar design to this study to assess whether
470 cranberry extract can help reduce antibiotic use for symptoms of acute, uncomplicated UTI.
471 The exploratory, underpowered analyses suggest that cranberry (alone and combined with
472 antibiotics) *may* have a beneficial impact on certain measures of antibiotic usage and
473 symptoms. These findings support the conduct of further, definitive research in this area.
474
475
476



For peer review only - http://bmjopen.bmj.com/site/about/guidelines.xhtml

477 **LIST OF ABBREVIATIONS**

478 CFU – Colony Forming Unit

479 GP – General Practitioner

480 HRA – Health Research Authority

481 MHRA - Medicines and Healthcare products Regulatory Agency

482 NDPCHS - Nuffield Department for Primary Care Health Sciences

483 PAC – Proanthocyanidin

484 REDCap - Research Electronic Data Capture

485 UPEC – Uropathogenic *Escherichia coli*

486 UTI – Urinary tract infection

487 **DECLARATIONS**

488 **Contributors:**

489 OAG was responsible for the research questions, writing the protocol, the design and running of the feasibility trial, data analysis and writing the manuscript. OAG is the guarantor of the manuscript. JA is a senior trial manager who assisted with running the feasibility trial and contributed to the manuscript. NW is a senior statistician who contributed to the protocol and statistical analysis plan, and verified statistical analyses of primary outcomes. CCB, MM, ADH and CJH helped to develop the research questions and study design and contributed to the manuscript. AB was a member of the TMG and contributed to the manuscript. The



For peer review only - http://bmjopen.bmj.com/site/about/guidelines.xhtml

corresponding author attests that all listed authors meet authorship criteria and that no others meeting the criteria have been omitted.

**Funding:**

This work was supported by the NIHR School for Primary Care Research (Grant reference number: SPCR-2014-10043). Indena S.p.A supplied Redicran capsules to the trial at no cost - they had no other role in the study. OAG's time is funded by the Wellcome Trust (Grant reference number: 203921). CJH received funding support from the NIHR SPCR Evidence Synthesis Working Group [project 390] and the NIHR Oxford BRC. AB receives funding from the NIHR School for Primary Care Research. The funders have no role in study design, manuscript submission, or collection, management, analysis or interpretation of study data. The views are those of the authors and not necessarily those of the NIHR or Department of Health and Social Care, nor the Wellcome Trust.

**Competing interests:**

All authors have completed the ICMJE uniform disclosure form at www.icmje.org/coi_disclosure.pdf and declare: authors had financial support from NIHR SPCR, the Wellcome Trust and the NIHR Oxford BRC for the submitted work; no financial relationships with any organisations that might have an interest in the submitted work in the previous three years; no other relationships or activities that could appear to have influenced the submitted work.

OAG receives funding from the NIHR SPCR and the Wellcome Trust. CCB is; a Senior Investigator of the National Institute of Health Research; Clinical Director of the University of Oxford Primary Care and Vaccines Collaborative Clinical Trials Unit; Clinical Director of the NIHR Oxford Community Medtech and Invitro diagnostics Cooperative, and; salaried



For peer review only - http://bmjopen.bmj.com/site/about/guidelines.xhtml

general practitioner for the Cwm Taf Morgannwg University Health Board. He has received funding from many public funding bodies for primary care research related to the management of common infections. He received payment for contributing to Advisory Boards for Pfizer in 2019, Roche Diagnostics in 2020, and for contributing to an Advisory Board for Janssen Pharmaceuticals about Respiratory Syncytial Virus treatment and vaccination from Janssen Pharmaceuticals in 2020, and holds an unrestricted grant form Janssen Pharmaceuticals for contributing to research on Respiratory Syncytial Virus. CJH reports he has received expenses and fees for his media work. He is Director of the CEBM at the University of Oxford, and Editor in Chief of BMJ Evidence-Based Medicine and an NIHR Senior Investigator. JA, NW, MM, ADH and AB have no Conflict of Interests to declare.

**Protocol:**

The protocol has been published and is freely available:

Gbinigie, O., Allen, J., Boylan, A. *et al.* Does cranberry extract reduce antibiotic use for symptoms of acute uncomplicated urinary tract infections (CUTI)? Protocol for a feasibility study. *Trials* **20,** 767 (2019). https://doi.org/10.1186/s13063-019-3860-z

**Ethics Approval:**

Informed consent was obtained from all participants prior to entry into the study. In accordance with the Declaration of Helsinki, the CUTI study was approved by the South



For peer review only - http://bmjopen.bmj.com/site/about/guidelines.xhtml

Central Oxford B Ethics Committee (REC reference: 18/SC/0673) and the Health Research Authority (IRAS Project ID: 249672).

**Transparency Statement:**

The lead author (the manuscript's guarantor) affirms that the manuscript is an honest, accurate, and transparent account of the study being reported; that no important aspects of the study have been omitted; and that any discrepancies from the study as originally planned (and, if relevant, registered) have been explained.

**Acknowledgements:**

The authors would like to thank all study participants, participating GP practices and staff in Oxford University's Primary Care Clinical Trials Unit who were responsible for electronic database development (Ms Rui Zhao and Mr Luis Castello).

The authors would also like to thank members of the Trial Steering Committee (TSC): Dr. Akke Vellinga (TSC Chair and epidemiologist); Ms. Julia Hamer-Hunt (PPI representative); Dr. Bernard Gudgin (PPI representative); Dr. Merlin Willcox (academic clinical lecturer and GP); Dr. Emma Thomas-Jones (research fellow and senior trials manager) and Dr. Kathy Hughes (clinical lecturer and GP).

We thank Dr Sarah Tonkin-Crine for helpful feedback on the manuscript, and PPI contributors for their involvement with the trial.

**Data sharing statement:**

Relevant anonymised data are available upon reasonable request made to the corresponding author.



For peer review only - http://bmjopen.bmj.com/site/about/guidelines.xhtml

**Patient consent for publication:**

Not applicable – no participant identifiable information included

## REFERENCES


1. Butler CC, Hillier S, Roberts Z, et al. Antibiotic-resistant infections in primary care are symptomatic for longer and increase workload: outcomes for patients with E. coli UTIs. 2006;56(530):686-92.
2. Little P, Merriman R, Turner S, et al. Presentation, pattern, and natural course of severe symptoms, and role of antibiotics and antibiotic resistance among patients presenting with suspected uncomplicated urinary tract infection in primary care: observational study. *BMJ* 2010;340:b5633.
3. Kronenberg A, Bütikofer L, Odutayo A, et al. Symptomatic treatment of uncomplicated lower urinary tract infections in the ambulatory setting: randomised, double blind trial. *bmj* 2017;359:j4784.
4. Moore M, Trill J, Simpson C, et al. Uva-ursi extract and ibuprofen as alternative treatments for uncomplicated urinary tract infection in women (ATAFUTI): a factorial randomized trial. 2019
5. Wagenlehner FM, Abramov-Sommariva D, Höller M, et al. Non-antibiotic herbal therapy (BNO 1045) versus antibiotic therapy (Fosfomycin Trometamol) for the treatment of acute lower uncomplicated urinary tract infections in women: a double-blind, parallel-group, randomized, multicentre, non-inferiority phase III trial. *J Urologia internationalis* 2018;101(3):327-36.
6. Gágyor I, Bleidorn J, Kochen MM, et al. Ibuprofen versus fosfomycin for uncomplicated urinary tract infection in women: randomised controlled trial. *bmj* 2015;351:h6544.
7. Vik I, Bollestad M, Grude N, et al. Ibuprofen versus pivmecillinam for uncomplicated urinary tract infection in women—A double-blind, randomized non-inferiority trial. *PLoS medicine* 2018;15(5):e1002569.




For peer review only - http://bmjopen.bmj.com/site/about/guidelines.xhtml

8. Butler CC, Hawking MK, Quigley A, et al. Incidence, severity, help seeking, and management of uncomplicated urinary tract infection: a population-based survey. *Br J Gen Pract* 2015;65(639):e702-e07.
9. Howell AB, Botto H, Combescure C, et al. Dosage effect on uropathogenic Escherichia coli anti-adhesion activity in urine following consumption of cranberry powder standardized for proanthocyanidin content: a multicentric randomized double blind study. 2010;10(1):94.
10. Howell AB, Reed JD, Krueger CG, et al. A-type cranberry proanthocyanidins and uropathogenic bacterial anti-adhesion activity. *Phytochemistry* 2005;66(18):2281-91.
11. Gbinigie O, Allen J, Boylan A-M, et al. Does cranberry extract reduce antibiotic use for symptoms of acute uncomplicated urinary tract infections (CUTI)? Protocol for a feasibility study. *Trials* 2019;20(1):1-9.
12. Watson L, Little P, Moore M, et al. Validation study of a diary for use in acute lower respiratory tract infection. *Family Practice* 2001;18(5):553-54.
13. National Institute for Health and Care Excellence. Urinary tract infection (lower): antimicrobial prescribing. NICE guideline [NG109] 2018 [Available from: https://www.nice.org.uk/guidance/ng109.
14. Foden N, Burgess C, Shepherd K, et al. A guide to the management of acute rhinosinusitis in primary care management strategy based on best evidence and recent European guidelines. 2013;63(616):611-13.
15. Julious SA. Sample size of 12 per group rule of thumb for a pilot study. *J Pharmaceutical Statistics: The Journal of Applied Statistics in the Pharmaceutical Industry* 2005;4(4):287-91.
16. Sim J, Lewis M. The size of a pilot study for a clinical trial should be calculated in relation to considerations of precision and efficiency. *Journal of clinical epidemiology* 2012;65(3):301-08.
17. Bleidorn J, Gágyor I, Kochen MM, et al. Symptomatic treatment (ibuprofen) or antibiotics (ciprofloxacin) for uncomplicated urinary tract infection?-results of a randomized controlled pilot trial. *BMC medicine* 2010;8(1):30.
18. Jepson RG, Williams G, Craig JC. Cranberries for preventing urinary tract infections. *The Cochrane Library* 2012

For peer review only



For peer review only - http://bmjopen.bmj.com/site/about/guidelines.xhtml

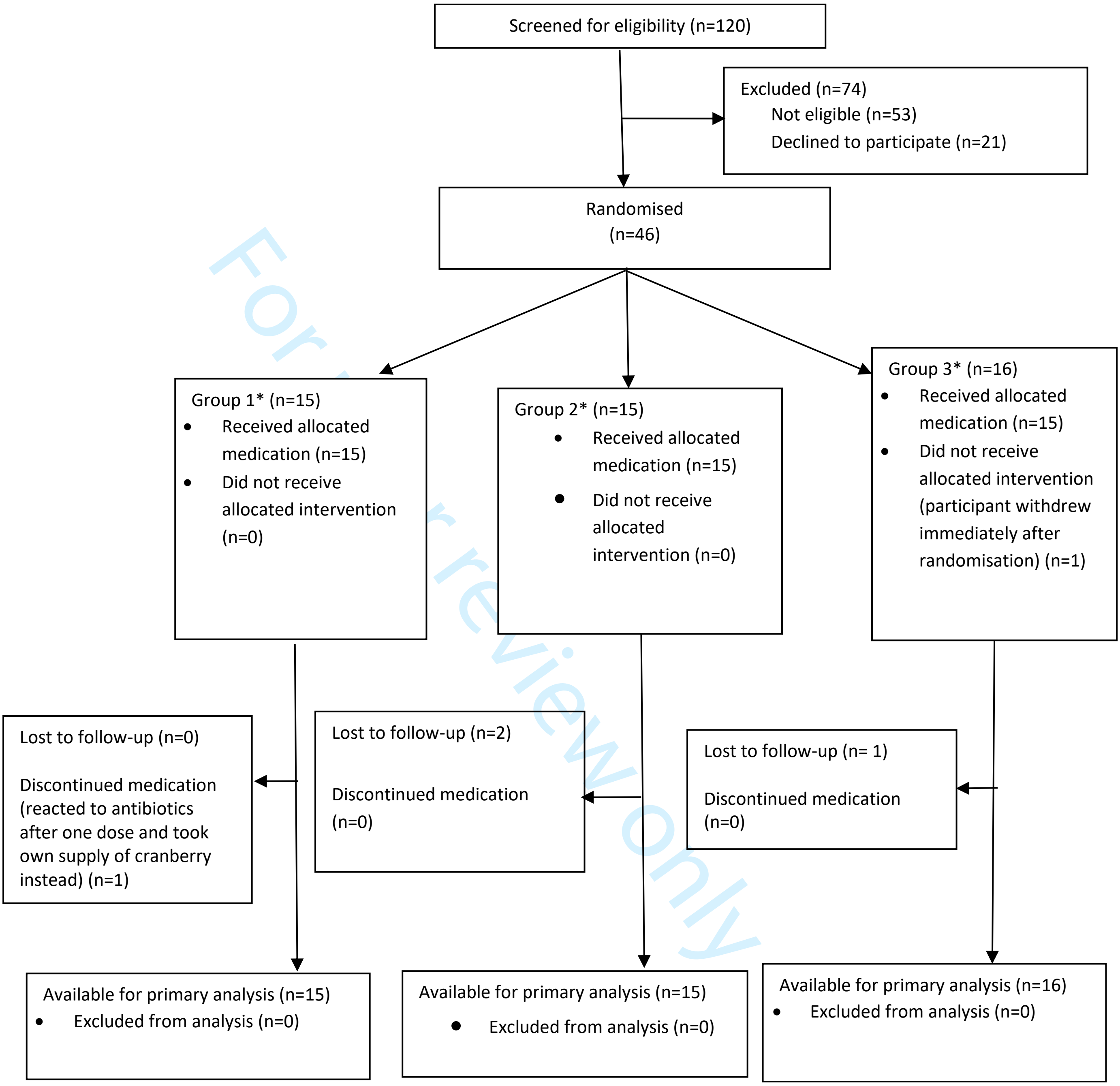


For peer review only

**Figure 1** – CONSORT diagram showing the flow of trial participants.
*Group 1 – Immediate antibiotics alone (control); Group 2 – Immediate antibiotics and immediate cranberry capsules; Group 3 – Immediate cranberry capsules and delayed antibiotics

**Supplementary file 1** – Estimate of the difference in proportions of adverse events between groups

| | Proportion (%) of participants experiencing an adverse event at 2 weeks [risk ratio (95% CI)] | Proportion (%) of participants experiencing an adverse event at 4 weeks [risk ratio (95% CI)] |
|---|---|---|
| **Group 1** (Control) n=15 | 1/15 (6.7%)<br><br>Missing: 0/15 | 2/15 (13.3%)<br><br>Missing: 0/15 |
| **Group 2** n=15 | 1/13 (7.7%)<br>[1.2 (0.1 to 16.7)]<br><br>Missing: 2/15 | 1/13 (7.7%)<br>[0.6 (0.1 to 5.7)]<br><br>Missing: 2/15 |
| **Group 3** n=16 | 3/13 (23.1%)<br>[3.5 (0.4 to 29.4)]<br><br>Missing: 3/16 | 3/13 (23.1%)<br>[1.7 (0.3 to 8.8 )]<br><br>Missing: 3/16 |

Group 1 – Immediate antibiotics alone (control); Group 2 – Immediate antibiotics and immediate cranberry; Group 3 – Immediate cranberry and delayed antibiotics. Group 2 and group 3 were compared with Group 1 (controls).

For peer review only

# CONSORT 2010 checklist of information to include when reporting a randomised trial*

| Section/Topic | Item No | Checklist item | Reported on page No |
|---|---|---|---|
| **Title and abstract** | | | |
| | 1a | Identification as a randomised trial in the title | 1 |
| | 1b | Structured summary of trial design, methods, results, and conclusions (for specific guidance see CONSORT for abstracts) | 2-3 |
| **Introduction** | | | |
| Background and objectives | 2a | Scientific background and explanation of rationale | 4 |
| | 2b | Specific objectives or hypotheses | 4 |
| **Methods** | | | |
| Trial design | 3a | Description of trial design (such as parallel, factorial) including allocation ratio | 5 |
| | 3b | Important changes to methods after trial commencement (such as eligibility criteria), with reasons | N/A |
| Participants | 4a | Eligibility criteria for participants | 5 |
| | 4b | Settings and locations where the data were collected | 5 |
| Interventions | 5 | The interventions for each group with sufficient details to allow replication, including how and when they were actually administered | 6 |
| Outcomes | 6a | Completely defined pre-specified primary and secondary outcome measures, including how and when they were assessed | 6-7 and protocol |
| | 6b | Any changes to trial outcomes after the trial commenced, with reasons | 7 |
| Sample size | 7a | How sample size was determined | 7 |
| | 7b | When applicable, explanation of any interim analyses and stopping guidelines | N/A |
| Randomisation: | | | |
| Sequence generation | 8a | Method used to generate the random allocation sequence | 7 |
| | 8b | Type of randomisation; details of any restriction (such as blocking and block size) | 7 |
| Allocation concealment mechanism | 9 | Mechanism used to implement the random allocation sequence (such as sequentially numbered containers), describing any steps taken to conceal the sequence until interventions were assigned | 7 |
| Implementation | 10 | Who generated the random allocation sequence, who enrolled participants, and who assigned participants to interventions | 7 |
| Blinding | 11a | If done, who was blinded after assignment to interventions (for example, participants, care providers, those | N/A |

For peer review only

| | | | |
|---|---|---|---|
| | | assessing outcomes) and how | |
| | 11b | If relevant, description of the similarity of interventions | N/A |
| Statistical methods | 12a | Statistical methods used to compare groups for primary and secondary outcomes | 8-9 |
| | 12b | Methods for additional analyses, such as subgroup analyses and adjusted analyses | N/A |
| **Results** | | | |
| Participant flow (a diagram is strongly recommended) | 13a | For each group, the numbers of participants who were randomly assigned, received intended treatment, and were analysed for the primary outcome | Figure 1 |
| | 13b | For each group, losses and exclusions after randomisation, together with reasons | Figure 1 |
| Recruitment | 14a | Dates defining the periods of recruitment and follow-up | 14 |
| | 14b | Why the trial ended or was stopped | 14 |
| Baseline data | 15 | A table showing baseline demographic and clinical characteristics for each group | Table 1 |
| Numbers analysed | 16 | For each group, number of participants (denominator) included in each analysis and whether the analysis was by original assigned groups | Figure 1 |
| Outcomes and estimation | 17a | For each primary and secondary outcome, results for each group, and the estimated effect size and its precision (such as 95% confidence interval) | Table 3 |
| | 17b | For binary outcomes, presentation of both absolute and relative effect sizes is recommended | Table 3 |
| Ancillary analyses | 18 | Results of any other analyses performed, including subgroup analyses and adjusted analyses, distinguishing pre-specified from exploratory | 24 and Table 3 |
| Harms | 19 | All important harms or unintended effects in each group (for specific guidance see CONSORT for harms) | 24 and Table 5 |
| **Discussion** | | | |
| Limitations | 20 | Trial limitations, addressing sources of potential bias, imprecision, and, if relevant, multiplicity of analyses | 26-27 |
| Generalisability | 21 | Generalisability (external validity, applicability) of the trial findings | 28-29 |
| Interpretation | 22 | Interpretation consistent with results, balancing benefits and harms, and considering other relevant evidence | 27-28 |
| **Other information** | | | |
| Registration | 23 | Registration number and name of trial registry | 3 |
| Protocol | 24 | Where the full trial protocol can be accessed, if available | 31-32 |
| Funding | 25 | Sources of funding and other support (such as supply of drugs), role of funders | 31 |

*We strongly recommend reading this statement in conjunction with the CONSORT 2010 Explanation and Elaboration for important clarifications on all the items. If relevant, we also recommend reading CONSORT extensions for cluster randomised trials, non-inferiority and equivalence trials, non-pharmacological treatments, herbal interventions, and pragmatic trials. Additional extensions are forthcoming: for those and for up to date references relevant to this checklist, see www.consort-statement.org.



For peer review only - http://bmjopen.bmj.com/site/about/guidelines.xhtml

# Does cranberry extract reduce antibiotic use for symptoms of acute uncomplicated urinary tract infections (CUTI)? A feasibility randomised trial.



| | |
|---|---|
| Journal: | *BMJ Open* |
| Manuscript ID | bmjopen-2020-046791.R1 |
| Article Type: | Original research |
| Date Submitted by the Author: | 25-Jan-2021 |
| Complete List of Authors: | Gbinigie, Oghenekome; University of Oxford, Nuffield Department of Primary Care Health Sciences<br>Allen, Julie; University of Oxford, Nuffield Department of Primary Care Health Sciences<br>Williams, Nicola; Nuffield Department of Primary Care Health Sciences, University of Oxford<br>Moore, Michael; University of Southampton Medical School, Primary Care Medical Group<br>Hay, Alastair; University of Bristol, Centre for Academic Primary Care<br>Heneghan, Carl; Oxford University, Primary Health Care<br>Boylan, Anne-Marie; Oxford University, Nuffield Department of Primary Care Health Sciences<br>Butler, Christopher C.; University of Oxford, |
| <b>Primary Subject Heading</b>: | Infectious diseases |
| Secondary Subject Heading: | General practice / Family practice |
| Keywords: | Urinary tract infections < UROLOGY, PRIMARY CARE, Clinical trials < THERAPEUTICS |
| | |



SCHOLARONE™ Manuscripts

*I, the Submitting Author has the right to grant and does grant on behalf of all authors of the Work (as defined in the below author licence), an exclusive licence and/or a non-exclusive licence for contributions from authors who are: i) UK Crown employees; ii) where BMJ has agreed a CC-BY licence shall apply, and/or iii) in accordance with the terms applicable for US Federal Government officers or employees acting as part of their official duties; on a worldwide, perpetual, irrevocable, royalty-free basis to BMJ Publishing Group Ltd ("BMJ") its licensees and where the relevant Journal is co-owned by BMJ to the co-owners of the Journal, to publish the Work in this journal and any other BMJ products and to exploit all rights, as set out in our licence.*

*The Submitting Author accepts and understands that any supply made under these terms is made by BMJ to the Submitting Author unless you are acting as an employee on behalf of your employer or a postgraduate student of an affiliated institution which is paying any applicable article publishing charge ("APC") for Open Access articles. Where the Submitting Author wishes to make the Work available on an Open Access basis (and intends to pay the relevant APC), the terms of reuse of such Open Access shall be governed by a Creative Commons licence – details of these licences and which Creative Commons licence will apply to this Work are set out in our licence referred to above.*

*Other than as permitted in any relevant BMJ Author's Self Archiving Policies, I confirm this Work has not been accepted for publication elsewhere, is not being considered for publication elsewhere and does not duplicate material already published. I confirm all authors consent to publication of this Work and authorise the granting of this licence.*

For peer review only

**Title:** Does cranberry extract reduce antibiotic use for symptoms of acute uncomplicated urinary tract infections (CUTI)? A feasibility randomised trial.

**Authors' names:** Oghenekome A Gbinigie[1] (0000-0002-2963-4491), Julie Allen[1], Nicola Williams[1], Michael Moore[2], Alastair D Hay[3], Carl J Heneghan[1], Anne-Marie Boylan[1], Christopher C Butler[1]

**Authors' addresses:**

[1] Nuffield Department of Primary Care Health Sciences, University of Oxford, Radcliffe Observatory Quarter, Woodstock Road, Oxford, OX2 6GG.

[2] Primary Care and Population Sciences, University of Southampton, SO16 5ST.

[3]Centre for Academic Primary Care, Population Health Sciences: Bristol Medical School, University of Bristol, BS8 2PS.

**Corresponding author:**

Dr Oghenekome Gbinigie

Nuffield Department of Primary Care Health Sciences, University of Oxford, Radcliffe Observatory Quarter, Woodstock Road, Oxford, OX2 6GG.

Email address: oghenekome.gbinigie@phc.ox.ac.uk

**Word count:** 4337

**Keywords:** Urinary Tract Infection, Vaccinium macrocarpon, General Practice, Primary Health Care, Clinical Trial



For peer review only - http://bmjopen.bmj.com/site/about/guidelines.xhtml

**Abstract**

**Objectives:** To determine the feasibility of conducting a randomised trial of the effectiveness of cranberry extract in reducing antibiotic use by women with symptoms of acute, uncomplicated urinary tract infection (UTI).

**Design:** Open-label feasibility randomised parallel group trial.

**Setting:** Four general practices in Oxfordshire

**Participants:** Women aged 18 years and above presenting to general practice with symptoms of acute, uncomplicated UTI.

**Interventions:** Women were randomly assigned using Research Electronic Data Capture (REDCap) in a 1:1:1 ratio to: 1) Immediate antibiotics alone (n=15); 2) Immediate antibiotics and immediate cranberry capsules for up to 7 days (n=15); or 3) Immediate cranberry capsules and delayed antibiotics for self-initiation in case of non-improvement or worsening of symptoms (n=16).

**Primary and secondary outcomes measures:** The primary outcome measures were: rate of recruitment of participants; numbers lost to follow-up; proportion of electronic diaries completed by participants; and acceptability of the intervention and study procedures to participants and recruiters. Secondary outcomes included an exploration of differences in symptom burden and antibiotic use between groups.

**Results:** Four GP practices (100%) were opened and recruited participants between 1st July and 2nd December 2019, with nine study participants recruited per month on average. 68.7% (46/67) of eligible participants were randomised (target 45) with a mean age of 48.4 years (SD 19.9, range 18 to 81). 89.1% (41/46) of diaries contained some participant entered data, and 69.6% (32/46) were fully complete. Three participants (6.5%) were lost to follow-up and two (4.4%) withdrew. Of women randomly assigned to take antibiotics alone (controls), one



For peer review only - http://bmjopen.bmj.com/site/about/guidelines.xhtml

third of respondents reported consuming cranberry products (33.3%, 4/12). There were no serious adverse events.

**Conclusions:** It appears feasible to conduct a randomised trial of the use of cranberry extract in the treatment of acute, uncomplicated UTI in general practice.

**Trial registration:** ISRCTN registry, ID: 10399299. Registered prospectively on 24th January 2019

**Strengths and limitations of this study**

- This feasibility trial addresses a novel use of cranberry extract in the treatment of *acute*, rather than prevention of *recurrent*, urinary tract infection.
- A pragmatic, open-label randomised trial design was employed.
- In keeping with pragmatic trials, no placebo control was used.

For peer review only

## Introduction

In light of rising levels of antimicrobial resistance, there has been growing interest in the use of non-antibiotic treatments for common bacterial infections, such as urinary tract infections (UTIs). UTIs are one of the commonest bacterial infections seen in primary care[1], and are nearly always treated with antibiotics.[2] Trials have evaluated non-antibiotic treatments for acute uncomplicated UTIs in primary care, [3-7] and use of ibuprofen has enabled some women to manage UTIs without taking antibiotics, albeit with a greater symptom burden compared with antibiotic treatment, and more cases of pyelonephritis.[3 6 7]

Cranberry has been extensively evaluated as a preventative agent for UTIs, with mixed results. Up to 27% of women report using cranberry to help treat the symptoms of an acute UTI. [8] Despite this, there is a lack of trials assessing whether cranberry can be used to treat the symptoms of *acute* UTIs, alone, or in combination with antibiotics. There is some mechanistic data to support this approach; urine collected within 6 hours of consumption of cranberry powder containing standardized amounts of Proanthocyanidins (PAC) has been shown to exert an anti-adhesion effect against Escherichia coli *in vitro*, in a dose-dependent fashion.[9] PAC with type A linkages, or the breakdown products after digestion in the gut, is believed to be the active ingredient in cranberry.[10]

The primary objective of this feasibility trial was therefore to determine the feasibility of conducting a randomised clinical trial to determine the effectiveness of cranberry extract in reducing antibiotic use by women with symptoms of acute, uncomplicated urinary tract infection (UTI) in general practice.



For peer review only - http://bmjopen.bmj.com/site/about/guidelines.xhtml

## Methods

Trial Design and Participants

This was an open-label, pragmatic, feasibility trial with randomisation in a 1:1:1 ratio between treatment groups in UK primary care. The eligibility criteria have been described in detail elsewhere.[11] In brief, women aged 18 years and above with symptoms of acute, uncomplicated UTI, to whom their healthcare practitioner would normally give a prescription for immediate antibiotics, and presenting to one of four participating General Practitioner (GP) practices in Oxfordshire, were eligible. Prospective participants had to be prepared to accept a delayed antibiotic prescription; that is, an antibiotic prescription with advice not to take the antibiotics unless their symptoms did not improve, or worsened. Women were excluded if they had signs of upper or complicated UTI, if they had received antibiotics in the preceding 7 days, if they had a symptom duration of 7 days or more, or if they regularly consumed cranberry products on five or more days per week.

Participants were emailed a link to an electronic symptom diary and were asked to complete it daily for 2 weeks. They were asked to rate a number of symptoms on a scale of 0 to 50, and to record any UTI treatments (study interventions or otherwise) that they were taking. The list of symptoms in the daily diary has been published with the feasibility trial protocol [11]. The scale used was an adaptation of a validated 7-point Likert scale developed by Watson *et al* [12]:

0 = Normal/not affected

1–9 = Very little problem

10–19 = Slight problem

20–29 = Moderately bad

30–39 = Bad



For peer review only - http://bmjopen.bmj.com/site/about/guidelines.xhtml

40–49 = Very bad

50 = As bad as it could be

We used this wider scale as part of the feasibility testing to see whether participants liked having more rating options. We also wondered whether the wider scale might enable better detection of within- and between-participant rating variation. A follow-up phone call took place at 2 weeks to encourage diary completion and obtain a minimum data set from participants whose diaries were incomplete. Women were invited for interview at a later date if they had provided consent at the point of trial recruitment to receive information about the interview study. A notes review took place at 4 weeks to record any clinical contacts that were believed to be related to the original UTI episode, as well as the urine culture result. Adverse event data were collected through symptom diary entries, through participant reports at the 2 week follow-up call, and through notes reviews.

<u>Interventions</u>

Women in Group 1 (control) received a prescription for first-line immediate antibiotics alone. In keeping with pragmatic trial design, the antibiotic prescribed and the duration was at the discretion of the recruiting clinician. In the UK, the usual recommended first-line treatment for acute uncomplicated UTI is a 3 day course of Nitrofurantoin.[13] Women assigned to Group 2 received an immediate prescription for first-line antibiotics and a course of cranberry capsules to be taken alongside antibiotics until symptom resolution, or up to a maximum of seven days. The cranberry capsules (Redicran) were supplied free to the study by Indena S.p.A. This company had no other involvement with the trial. Each capsule contained 18 mg of PAC. Women were advised to take two capsules twice daily at 12 hour intervals, making a total of 72 mg of PAC daily. Ex-vivo studies suggest that consumption of 72 mg PAC has anti-adhesion activity against Uropathogenic Escherichia coli (UPEC). [9] Women in Group 3

For peer review only

were advised to take immediate cranberry capsules (Redicran) for up to seven days. They also received a delayed antibiotic prescription to be used if their symptoms did not improve within 3-5 days, or if their symptoms worsened.

Outcomes

The primary outcomes were feasibility measures,[11] namely, the rate of recruitment of participants, the number of participants lost to follow-up, and the acceptability of trial processes and the quality of data capture using electronic symptom diaries. These data can contribute to sample size calculations for an adequately powered trial, and additionally increase the chance of such a trial being successful. The study was not powered to detect differences between groups. Nevertheless, secondary outcomes included an exploration of between group differences in measures of symptom burden, antibiotic consumption and adverse events.[11]

Although not made explicit in the protocol, a comparison of the time to feeling fully recovered between groups was made, as pre-specified in the statistical analysis plan. These data were already being collected through the electronic diary, which had received ethical approval. A decision to conduct an exploratory analysis of the proportion of participants experiencing ‘double sickening’ (feeling fully recovered and then subsequently feeling unwell within 2 weeks of trial enrolment) was made after the statistical analysis plan had been completed, but before the trial data had been reviewed or analyses commenced. The concept of double sickening has been noted in acute respiratory illness; [14]we felt that an exploratory analysis of this concept in the context of acute UTI would be novel and useful. A substantial amendment was approved by the ethics committee after trial recruitment had started to allow the trial team to interview enrolling clinicians to the trial, as part of the study’s feasibility assessment.



For peer review only - http://bmjopen.bmj.com/site/about/guidelines.xhtml

Sample size and randomization

A sample size of 45 participants was chosen to enable estimation of a loss-to-follow-up rate of 20% with a confidence interval of 9.6 to 34.6%.[11] Sample sizes of between 24[15] and 50[16] have been suggested for pilot studies.

The randomisation sequence was generated using Stata v15.1 (StataCorp LP, College station, TX) by a statistician at the Nuffield Department of Primary Care Health Sciences, University of Oxford, using computer generated block randomisation with variable block size. The sequence was password-protected and accessible only by the statistician. Participants were randomised by recruiting clinicians through the click of a button on the electronic trial registration page, which was created using Research Electronic Data Capture (REDCap).

Statistical methods

Unless otherwise stated, primary outcomes are presented as proportions and percentages for each trial group and for the entire trial population. Participants in group 2 and group 3 were compared separately with participants in group 1 (the control group).

The number of antibiotic courses (or part thereof) consumed by participants in each group at 2 weeks and 4 weeks was compared using Poisson regression and reported using incidence rate ratios. The difference in the duration of symptoms rated moderately bad or worse between groups was compared using negative binomial regression; incidence rate ratios are reported and a Kaplan Meier graph plotted (supplementary Figure). The mean symptom burden scores (from day 2 to day 4 inclusive) were compared between groups using linear regression, and reported using mean differences. The difference in time to feeling fully recovered between groups was compared using the Cox proportional-hazards model; hazard

For peer review only



For peer review only - http://bmjopen.bmj.com/site/about/guidelines.xhtml

ratios are presented and a Kaplan Meier graph was plotted (Supplementary Figure). Each estimate was adjusted for study site.

A risk ratio and 95% confidence interval was used to estimate the difference in the proportion of participants consuming a course of antibiotics (or part thereof) between groups at two weeks and four weeks, the difference in the proportion of adverse events between groups at both two weeks and four weeks, and to compare the proportion of participants in each group experiencing double sickening. It was not possible to use log binomial regression, as originally planned, to compare the proportion of participants consuming antibiotics between groups as the model would not converge. A risk ratio and 95% confidence interval was used in lieu of Fisher's exact test (as originally planned) to estimate the difference in proportion of adverse events between groups in order to generate 95% confidence intervals rather than p values. As this study was not powered to detect differences between groups, we had pre-specified that p values would not be reported.

Intention to treat analysis was conducted for all analyses and estimates are presented with their 95% confidence intervals. Due to the small sample size and the fact that this is a feasibility study, no subgroup or sensitivity analyses were conducted.

<u>Patient and Public Involvement (PPI)</u>

Four PPI contributors have been closely involved with the study from the outset, two of whom sit on the Trial Steering Committee. During trial conception and development, they confirmed that the topic chosen was important. They critiqued all public facing trial documentation to ensure that it was free of jargon, and their feedback was incorporated. They were also involved in the design of the electronic diary, helping to ensure that it was user-friendly. PPI contributors unanimously felt that the diary would not be onerous for participants; and suggested it may make the experience of participating in the trial more



For peer review only - http://bmjopen.bmj.com/site/about/guidelines.xhtml

For peer review only

meaningful, stating "…completing the symptom diary hooks people in. It makes them feel that they are contributing." The results of the study have been shared with PPI contributors, allowing them to give their views on ways to explain the findings, which have been incorporated into the discussion. PPI contributors are also helping to inform the dissemination of the study findings.

## Results

Baseline data

Baseline information was completed electronically by participants upon accessing the electronic diary on day one of enrolment, prior to inputting their day one symptom ratings. The mean age of participants was 48.3 years (range: 18 to 81 years). The mean age of women in the immediate cranberry and delayed antibiotics group was lower than the other two groups. Most women had experienced a previous UTI (85.4%, 35/41). 75.6% (31/41) of respondents were expecting antibiotics. 61% (25/41) believed that cranberry could help their symptoms, whilst 24.4% (10/41) believed that their symptoms could improve without antibiotics (table 1).

**Table 1** – Baseline Characteristics of Participants

| Proportion (%), unless otherwise specified | Group 1 (n=15) | Group 2 (n=15 ) | Group 3 (n=16) | Total (n=46 ) |
|---|---|---|---|---|
| **Age in years**, Mean (SD) [range] | 52.4 | 52.4 | 40.8 | 48.3 |



For peer review only - http://bmjopen.bmj.com/site/about/guidelines.xhtml

| | | | | |
|---|---|---|---|---|
| | (21.4) [18-81] | (22.4) [19-81] | (14.0) [20-63] | (19.9) [18-81] |
| **Previous UTI** | | | | |
| Yes | 12/15 (80%) | 12/13 (92.3%) | 11/13 (84.6%) | 35/41 (85.4%) |
| No | 3/15 (20%) | 1/13 (7.7%) | 2/13 (15.4%) | 6/41 (14.6) |
| Missing | 0/15 | 2/15 | 3/16 | 5/46 |
| **Previous UTI in the past year** | | | | |
| Yes | 7/15 (46.7%) | 6/13 (46.2%) | 6/13 (46.2%) | 19/41 (46.3%) |
| No | 5/15 (33.3%) | 6/13 (46.2%) | 5/13 (38.5%) | 16/41 (39.0%) |
| N/A *(Have never had a previous UTI)* | 3/15 (20%) | 1/13 (7.7%) | 2/13 (15.4%) | 6/41 (14.6%) |
| Missing | 0/15 | 2/15 | 3/16 | 5/46 |
| **If had a UTI in the past year, number of months since last UTI – mean (SD), median [range]** | 5.4 (4.5), 4 [0-12] n=7 | 5 (4.2), 5 [0-11] n=5 | 7 (3.6), 7 [3-12] n=5 | 5.8 (4.2), 5 [0-12] n=17 |
| Can't remember | 0/15 (0%) | 1/13 (7.7%) | 1/12 (8.3%) | 2/40 (5%) |
| Not applicable | 8/15 (53.3%) | 7/13 (53.9%) | 6/12 (50%) | 21/40 (52.5%) |
| Missing | 0/15 | 2/15 | 4/16 | 6/46 |
| **Number of UTIs in the past year:** | | | | |
| Zero | 5/15 (33.3%) | 7/13 (53.9%) | 4/13 (30.8%) | 16/41 (39.0%) |
| One | 3/15 (20%) | 2/13 (15.4%) | 5/13 (38.5%) | 10/41 (24.4%) |
| Two | 2/15 (13.3%) | 2/13 (15.4%) | 1/13 (7.7%) | 5/41 (12.2%) |
| Three or more | 2/15 (13.3%) | 1/13 (7.7%) | 1/13 (7.7%) | 4/41 (9.8%) |
| Not applicable *(Have never had a previous UTI)* | 3/15 (20%) | 1/13 (7.7%) | 2/13 (15.4%) | 6/41 (14.6%) |
| Missing | 0/15 | 2/15 | 3/16 | 5/46 |
| **Were you expecting antibiotic treatment?** | | | | |
| Yes | 13/15 (86.7%) | 10/13 (76.9%) | 8/13 (61.5%) | 31/41 (75.6%) |
| No | 0/15 (0%) | 1/13 (7.7%) | 1/13 (7.7%) | 2/41 (4.9%) |
| Unsure | 2/15 (13.3%) | 2/13 (15.4%) | 4/13 (30.8%) | 8/41 (19.5%) |
| Missing | 0/15 | 2/15 | 3/16 | 5/46 |
| **Were you expecting tests/investigations?** | | | | |
| Yes | 12/15 (80%) | 10/13 (76.9%) | 8/13 (61.5%) | 30/41 (73.2%) |



For peer review only - http://bmjopen.bmj.com/site/about/guidelines.xhtml

| | | | | |
|---|---|---|---|---|
| No | 3/15 (20%) | 3/13 (23.1%) | 4/13 (30.8%) | 10/41 (24.4%) |
| Unsure | 0/15 (0%) | 0/13 (0%) | 1/13 (7.7%) | 1/41 (2.4%) |
| Missing | 0/15 | 2/15 | 3/16 | 5/46 |
| **Were you expecting advice?** | | | | |
| Yes | 12/15 (80%) | 11/12 (91.7%) | 13/13 (100%) | 36/40 (90%) |
| No | 2/15 (13.3%) | 1/12 (8.3%) | 0/13 (0%) | 3/40 (7.5%) |
| Unsure | 1/15 (6.7%) | 0/12 (0%) | 0/13 (0%) | 1/40 (2.5%) |
| Missing | 0/15 | 3/15 | 3/16 | 6/46 |
| **Were you expecting something else?** | | | | |
| Yes | 0/15 (0%) | 1/13 (7.7%) - *Information and prescription (1 participant)* | 0/13 (0%) | 1/41 (2.4%) |
| No | 15/15 (100%) | 10/13 (76.9%) | 11/13 (84.6%) | 36/41 (87.8%) |
| Unsure | 0/15 (0%) | 2/13 (15.4%) | 2/13 (15.4%) | 4/41 (9.8%) |
| Missing | 0/15 | 2/15 | 3/16 | 5/46 |
| **Was your last UTI treated with antibiotics?** | | | | |
| Yes | 8/15 (53.3%) | 9/13 (69.2%) | 10/13 (76.9%) | 27/41 (65.9%) |
| No | 4/15 (26.7%) | 3/13 (23.1%) | 1/13 (7.7%) | 8/41 (19.5%) |
| Not applicable | 3/15 (20%) | 1/13 (7.7%) | 2/13 (15.4%) | 6/41 (14.6%) |
| Missing | 0/15 | 2/15 | 3/16 | 5/46 |
| **Was your last UTI treated with something other than antibiotics?** | | | | |
| Yes | 2/15 (13.3%) - *Cystitis sachets (2 participants)* | 1/13 (7.7%) - *Cystitis sachets (1 participant)* | 0/13 (0%) | 3/41 (7.3%) |
| No | 10/15 (66.7%) | 11/13 (84.6%) | 11/13 (84.6%) | 32/41 (78.1%) |
| Not applicable | 3/15 (20%) | 1/13 (7.7%) | 2/13 (15.4%) | 6/41 (14.6%) |
| Missing | 0/15 | 2/15 | 3/16 | 5/46 |
| **Did you receive no treatment for your last UTI?** | | | | |



For peer review only - http://bmjopen.bmj.com/site/about/guidelines.xhtml

| | | | | |
|---|---|---|---|---|
| Yes | 1/15 (6.7%) | 0/13 (0%) | 0/13 (0%) | 1/41 (2.4%) |
| No | 11/15 (73.3%) | 12/13 (92.3%) | 11/13 (84.6%) | 34/41 (82.9%) |
| Not applicable | 3/15 (20%) | 1/13 (7.7%) | 2/13 (15.4%) | 6/41 (14.6%) |
| Missing | 0/15 | 2/15 | 3/16 | 5/46 |
| **Unable to remember how last UTI was treated** | | | | |
| Yes | 1/15 (6.7%) | 1/13 (7.7%) | 1/13 (7.7%) | 3/41 (7.3%) |
| No | 11/15 (73.3%) | 11/13 (84.6%) | 10/13 (76.9%) | 32/41 (78.1%) |
| Not applicable | 3/15 (20%) | 1/13 (7.7%) | 2/13 (15.4%) | 6/41 (14.6%) |
| Missing | 0/15 | 2/15 | 3/16 | 5/46 |
| **Treatments tried before consulting primary care** | | | | |
| *Cranberry products* | | | | |
| Yes | 2/15 (13.3%) | 3/13 (23.1%) | 3/13 (23.1%) | 8/41 (19.5%) |
| No | 13/15 (86.7%) | 10/13 (76.9%) | 10/13 (76.9%) | 33/41 (80.5%) |
| Missing | 0/15 | 2/15 | 3/16 | 5/46 |
| *Other fruit juice* | | | | |
| Yes | 1/15 (93.3%) | 1/13 (7.7%) | 0/13 (0%) | 2/41 (4.9%) |
| No | 14/15 (93.3%) | 12/13 (92.3%) | 13/13 (100%) | 39/41 (95.1%) |
| Missing | 0/15 | 2/15 | 3/16 | 5/46 |
| *Bicarbonate solution* | | | | |
| Yes | 0/15 (0%) | 0/13 (0%) | 0/13 (0%) | 0/41 (0%) |
| No | 15/15 (100%) | 13/13 (100%) | 13/13 (100%) | 41/41 (100%) |
| Missing | 0/15 | 2/15 | 3/16 | 5/46 |
| *Sodium/potassium citrate (cystitis sachets)* | | | | |
| Yes | 3/15 (20%) | 7/13 (53.9%) | 1/13 (7.7%) | 11/41 (26.8%) |
| No | 12/15 (80%) | 6/13 (46.2%) | 12/13 (92.3%) | 30/41 (73.2%) |
| Missing | 0/15 | 2/15 | 3/16 | 5/46 |
| *Uvacin/Uva ursi (bearberry)* | | | | |
| Yes | 0/15 (0%) | 0/13 (0%) | 0/13 (0%) | 0/41 (0%) |
| No | 15/15 (100%) | 13/13 (100%) | 13/13 (100%) | 41/41 (100%) |
| Missing | 0/15 | 2/15 | 3/16 | 5/46 |



For peer review only - http://bmjopen.bmj.com/site/about/guidelines.xhtml

| | | | | |
|---|---|---|---|---|
| *Pain relief (e.g. paracetamol/Ibuprofen)* | | | | |
| Yes | 6/15 (40%) | 7/13 (53.9%) | 4/13 (30.8%) | 17/41 (41.5%) |
| No | 9/15 (60%) | 6/13 (46.2%) | 9/13 (69.2%) | 24/41 (58.5%) |
| Missing | 0/15 | 2/15 | 3/16 | 5/46 |
| *Other treatment used?* | | | | |
| Yes | 2/15 (13.3%) – *Water (2 participants)* | 2/13 (15.4%) – *Tea tree in bath (1 participant) Sachets (1 participant)* | 2/13 (15.4%) – *Water (2 participants)* | 6/41 (14.6%) |
| No | 13/15 (86.7%) | 11/13 (84.6%) | 11/13 (84.6%) | 35/41 (85.4%) |
| Missing | 0/15 | 2/15 | 3/16 | 5/46 |
| *None* | | | | |
| Yes | 7/15 (46.7%) | 2/13 (15.4%) | 5/13 (38.5%) | 14/41 (34.2%) |
| No | 8/15 (53.3%) | 11/13 (84.6%) | 8/13 (61.5%) | 27/41 (65.9%) |
| Missing | 0/15 | 2/15 | 3/16 | 5/46 |
| **Number of days symptomatic** | | | | |
| 0 days | 1/15 (6.7%) | 2/13 (15.4%) | 1/13 (7.7%) | 4/41 (9.8%) |
| 1 day | 3/15 (20%) | 1/13 (7.7%) | 2/13 (15.4%) | 6/41 (14.6%) |
| 2 days | 4/15 (26.7%) | 3/13 (23.1%) | 5/13 (38.5%) | 12/41 (29.3%) |
| 3 days | 2/15 (13.3%) | 2/13 (15.4%) | 4/13 (30.8%) | 8/41 (19.5%) |
| >3 days | 5/15 (33.3%) | 5/13 (38.5%) | 1/13 (7.7%) | 11/41 (26.8%) |
| Missing | 0/15 | 2/15 | 3/16 | 5/46 |
| **Belief that cranberry can help symptoms** | | | | |
| Yes | 10/15 (66.7%) | 7/13 (53.9%) | 8/13 (61.5%) | 25/41 (61%) |
| No | 1/15 (6.7%) | 0/13 (0%) | 0/13 (0%) | 1/41 (2.4%) |
| Unsure | 4/15 (26.7%) | 6/13 (46.2%) | 5/13 (38.5%) | 15/41 (36.6%) |
| Missing | 0/15 | 2/15 | 3/16 | 5/46 |
| **Belief that symptoms can get better without antibiotics** | | | | |
| Yes | 5/15 (33.3%) | 1/13 (7.7%) | 4/13 (30.8%) | 10/41 (24.4%) |



For peer review only - http://bmjopen.bmj.com/site/about/guidelines.xhtml

| | | | | |
|---|---|---|---|---|
| No | 4/15 (26.7%) | 4/13 (30.8%) | 2/13 (15.4%) | 10/41 (24.4%) |
| Unsure | 6/15 (40%) | 8/13 (61.5%) | 7/13 (53.9%) | 21/41 (51.2%) |
| Missing | 0/15 | 2/15 | 3/16 | 5/46 |

Group 1 – Immediate antibiotics alone (control); Group 2 – Immediate antibiotics and immediate cranberry; Group 3 – Immediate cranberry and delayed antibiotics.

## Primary Outcomes

Forty-six women were recruited to the trial, surpassing the target of 45 (See figure 1). Of the 21 women who were eligible but declined to take part, the most common reason for declining was not wishing to be randomised to receive delayed antibiotics (42.9%, 9/21). The first participant was recruited on 11th July 2019 and the final participant was recruited on 2nd December 2019, with the final follow-up taking place on 30th December 2019. The findings of qualitative interviews with 14 trial participants and eight trial recruiters will be presented elsewhere.

89.1% (41/46) of electronic diaries had some data entered by participants, whilst 69.6% (32/46) of electronic diaries were fully complete (table 2). There were no electronic diary failures necessitating the issue of paper diaries. A mean of 9.2 participants were recruited per month, which exceeded predicted recruitment of between four and eight participants each month. All GP practices that were invited to recruit to the trial agreed to participate (100%, 5/5), however, one GP practice withdrew prior to site opening as they predicted that they would no longer have capacity to support the trial.

**Table 2** – Primary Outcomes

| Proportion (%), unless otherwise specified | Group 1 (n=15) | Group 2 (n=15 ) | Group 3 (n=16) | Total (n=46 ) |
|---|---|---|---|---|



For peer review only - http://bmjopen.bmj.com/site/about/guidelines.xhtml

| | | | | |
|---|---|---|---|---|
| **Number of diaries with some data points entered by participant by end of participant follow-up** | 15 (100%) | 13 (86.7%) | 13 (81.3%) | 41 (89.1%) |
| **Number of diaries fully completed by participant by end of participant follow-up** | 13 (86.7%) | 10 (66.7%) | 9 (56.3%) | 32 (69.6%) |
| **Paper diaries issued due to failure of electronic diary** | 0 (0%) | 0 (0%) | 0 (0%) | 0 (0%) |
| **Number randomised as a proportion of the target recruitment number** | 46/45 (102.2%) | | | |
| **Number randomised as a proportion of invited participants** | 46/120 (38.3%) | | | |
| **Number randomised as a proportion of eligible participants** | 46/67 (68.7%) | | | |
| No. patients invited | | | | 120 |
| No. patients eligible | | | | 67 |
| No. participants enrolled | 15 | 15 | 16 | 46 |
| No. participants randomised | 15 | 15 | 16 | 46 |
| No. recruited per month (n) | | | | 9.2 |
| **Number of participants lost to follow-up** | 0 (0%) | 2 (13.3%) | 1 (6.3%) | 3 (6.5%) |
| **Number of participants withdrawing from study** | 0 (0%) | 0 (0%) | 2 (12.5%) | 2 (4.4%) |
| **Side effects or problems related to cranberry consumption** | | | | |
| Yes | 0/13 (0%) | 0/10 (0%) | 1/11 (9.1%) | 1/34 (2.9%) |
| No | 1/13 (7.7%) | 10/10 (100%) | 10/11 (90.9%) | 21/34 (61.8%) |
| Not applicable | 12/13 (92.3%) | 0/10 (0%) | 0/11 (0%) | 12/34 (35.3%) |
| Missing | 2/15 | 5/15 | 5/16 | 12/46 |
| **Problems completing the electronic diary** | | | | |
| No problem | 14/14 (100%) | 9/10 (90%) | 9/11 (81.8%) | 32/35 (91.4%) |
| Some problems | 0/14 (0%) | 1/10 (10%) | 2/11 (18.2%) | 3/35 (8.6%) |
| Lots of problems | 0/14 (0%) | 0/10 (0%) | 0/11 (0%) | 0/35 (0%) |
| Missing | 1/15 | 5/15 | 5/16 | 11/46 |
| **Problems using the scale of 0-50** | | | | |
| No problem | 12/14 (85.7%) | 9/9 (100%) | 10/12 (83.3%) | 31/35 (88.6%) |
| Some problems | 2/14 (14.3%) | 0/9 (0%) | 2/12 (16.7%) | 4/35 (11.4%) |



For peer review only - http://bmjopen.bmj.com/site/about/guidelines.xhtml

| | | | | |
|---|---|---|---|---|
| Lots of problems | 0/14 (0%) | 0/9 (0%) | 0/12 (0%) | 0/35 (0%) |
| Missing | 1/15 | 6/15 | 4/16 | 11/46 |
| **Proportion of invited GP practices agreeing to participate in the trial** | 5/5 (100%) | | | |
| **Proportion of invited GP practices opened and recruiting to the trial** | 4/5 (80%) | | | |
| **Acceptability of the study to participants and recruiters, participants' experience of having a UTI and thoughts on self-help for UTIs.** | Interviews with trial participants and recruiters will be reported elsewhere. | | | |

Group 1 – Immediate antibiotics alone (control); Group 2 – Immediate antibiotics and immediate cranberry; Group 3 – Immediate cranberry and delayed antibiotics.

Only one participant reported a side effect or problem related to consuming cranberry capsules in the electronic diary (table 2). Few participants reported experiencing problems completing the electronic diary (8.6%, 3/35) or rating their symptoms using the scale of 0 to 50 (11.4%, 4/35).

Three participants were lost to follow-up (Figure 1). Loss to follow-up was determined to have occurred when a participant did not make any electronic diary entries and it was not possible to contact them at the 2 week follow-up call.

Two participants withdrew from the study. One participant was consented and randomly assigned to immediate cranberry capsules with delayed antibiotics. As soon as she received this assignment, she decided to withdraw from the study and did not receive the cranberry capsules. Another participant was consented and also randomised to immediate cranberry and delayed antibiotics. The day after being enrolled, she emailed the trial team asking to be removed from the mailing list (links to the electronic diary were sent by email). In light of this, the chief investigator withdrew the participant from the study.

For peer review only



For peer review only - http://bmjopen.bmj.com/site/about/guidelines.xhtml

Participants were able to provide free-text comments on the final day (day 14) of diary completion. Free-text boxes allowed women to provide general comments about the trial, as well as comments specifically about difficulties using the electronic diary, difficulties using the rating scale and any problems related to consuming cranberry capsules. Fourteen women provided comments.

Some women provided general feedback, including being "happy to help" with the study and finding the diary "very easy to complete." Two participants described some difficulty keeping up with filling in the diary *"Just time and keeping up with it that is all. Happy to have taken part."* (41-8, Immediate cranberry and delayed antibiotics). Another participant mentioned that she did not receive a link to complete the final set of diary questions, and one woman was unclear about the guidance regarding the need to continue completing the diary once symptoms had resolved for two consecutive days. Two women commented that the scale of 0 to 50 was "too wide a range."

One woman stated that she was sceptical about cranberry at first, but found that it "helped a lot" (32-11, Immediate cranberry and delayed antibiotics). One participant found that the combination of antibiotic with cranberry cleared her infection "quite quickly" (32-14, Immediate antibiotics and immediate cranberry), whilst another found that the antibiotic/cranberry combination resulted in her taking a shorter course of antibiotics than usual – *"Nice to only take three days of antibiotics not a week"* (23-3, Immediate antibiotics and immediate cranberry). One woman stated that despite starting with cranberry, she had to take antibiotics on day 2 as she was in pain (41-2, Immediate cranberry and delayed antibiotics).

Three adverse events were picked up from free-text comments (see Harms section below).

**Secondary Outcomes**



For peer review only - http://bmjopen.bmj.com/site/about/guidelines.xhtml

Approximately one third of respondents reported contacting a healthcare practitioner within two weeks of being enrolled (36.4%, 12/33). Most of these women contacted their GP practice (GP or nurse at their surgery) - one woman reported liaising with a pharmacist, and another contacted an out of hours GP. No participants reported requiring Accident and Emergency or secondary care input. Just over half (54.3%, 25/46) of the urine samples at the time of enrolment were positive (organism growth greater than $10^5$/CFU).

Exploratory analyses of effectiveness were conducted, whilst recognising that the study was not powered for these analyses (table 3). For this reason, p values have not been reported and any differences detected between groups are inconclusive.

For peer review only



For peer review only - http://bmjopen.bmj.com/site/about/guidelines.xhtml

**Table 3** – Exploratory effectiveness outcomes

| | Proportion (%) of participants consuming a course of antibiotics (or part thereof) at 2 weeks [risk ratio (95% CI)]‡ | Proportion (%) of participants consuming a course of antibiotics (or part thereof) at 4 weeks [risk ratio (95% CI)]‡ | Mean (SD) number of courses of antibiotics consumed at 2 weeks [adjusted incidence rate ratio (95% CI)] ‡† | Mean (SD) number of courses of antibiotics consumed at 4 weeks [adjusted incidence rate ratio (95% CI)] ‡† | Median (IQR) number of days of symptoms rated moderately bad or worse [adjusted incidence rate ratio (95% CI)] ‡† | Mean (SD) symptom burden from day 2 to 4 inclusive [adjusted mean difference (95% CI)] ‡†¥ | Median (IQR) days to feeling fully recovered [adjusted hazard ratio (95% CI)] ‡† | Proportion (%) of participants experiencing double sickening up to 2 weeks [risk ratio (95% CI)]‡ |
|---|---|---|---|---|---|---|---|---|
| **Group 1** (Control) n=15 | 15/15 (100%)<br><br>Missing: 0/15 | 15/15 (100%)<br><br>Missing: 0/15 | 1.5 (0.5)<br><br>Missing: 0/15 | 1.6 (0.6)<br><br>Missing: 0/15 | 2 (1 to 2)<br><br>Missing: 0/15 | 6.3 (3.4)<br><br>Missing: 1/15 | 5 (4 to 6)<br><br>Missing: 0/15 | 6/14 (42.9%)<br><br>Missing: 1/15 |
| **Group 2** n=15 | 13/13 (100%)<br>[1 (1 to 1)]<br><br>Missing: 2/15 | 13/13 (100%)<br>[1 (1 to 1)]<br><br>Missing: 2/15 | 1.1 (0.3)<br>[0.7 (0.4 to 1.4 )]<br><br>Missing: 2/15 | 1.2 (0.4)<br>[0.7 (0.4 to 1.4)]<br><br>Missing: 2/15 | 2 (1 to 3)<br>[1.2 (0.7 to 2.1)]<br><br>Missing: 2/15 | 6.2 (8.0)<br>[0.6 (-4.0 to 5.3)]<br><br>Missing: 4/15 | 4 (3 to 6)<br>[1.7 (0.7 to 4.1)]<br><br>Missing: 2/15 | 3/11 (27.3%)<br>[0.6 (0.2 to 2.0)]<br><br>Missing: 4/15 |
| **Group 3** n=16 | 11/14 (78.6%)<br>[0.8 (0.6 to 1.0]<br><br>Missing: 2/16 | 11/14 (78.6%)<br>[0.8 (0.6 to 1.0]<br><br>Missing: 2/16 | 0.9 (0.6)<br>[0.6 (0.3 to 1.2 )]<br><br>Missing: 2/16 | 0.9 (0.6)<br>[0.6 (0.3 to 1.2)]<br><br>Missing: 2/16 | 4 (2 to 8)<br>[2.9 (1.7 to 5.1)]<br><br>Missing: 4/16 | 11.2 (8.5)<br>[7.9 (2.6 to 13.2)]<br><br>Missing: 7/16 | 13 (5 to 9)<br>[0.6 (0.2 to 1.4)]<br><br>Missing: 3/16 | 0/12 (0%)<br>[0 (N/A)]<br><br>Missing: 4/16 |

Group 1 – Immediate antibiotics alone (control); Group 2 – Immediate antibiotics and immediate cranberry; Group 3 – Immediate cranberry and delayed antibiotics.

‡Group 2 and group 3 were compared with Group 1 (controls).

†Model adjusted for site

¥ The mean symptom burden for each participant was calculated by summing the total ratings for all symptoms between day 2 and day 4 (inclusive) and dividing this by the number of symptom ratings made by the participant between day 2 and day 4 (inclusive).

**Abbreviations:** SD: Standard Deviation; %: Percentage



For peer review only - http://bmjopen.bmj.com/site/about/guidelines.xhtml

There was a 20% reduction in the risk of consuming a course of antibiotics (or part thereof) in the immediate cranberry group (group 3), compared with the immediate antibiotics alone group (group 1- controls). This was the case at both 2 weeks and 4 weeks (risk ratio 0.8; 95% CI: 0.6 to 1.0). The number of courses of antibiotics consumed at 2 weeks and at 4 weeks was reduced in groups 2 (immediate antibiotics and immediate cranberry) and 3 (immediate cranberry and delayed antibiotics), compared with the control group (adjusted incidence rate ratios: 0.7; 95% CI: 0.4 to 1.4 and 0.6; 95% CI: 0.3 to 1.2 for group 2 and group 3, respectively). Participants in groups 2 and 3 had a longer duration of symptoms rated moderately bad or worse, compared with those in the control group (adjusted incidence rate ratios: 1.2; 95% CI: 0.7 to 2.1 and 2.9; 95% CI 1.7 to 5.1 for group 2 and group 3, respectively). The mean symptom burden (from day 2 to day 4 inclusive) was reduced in group 2 participants (adjusted mean difference: 0.6; 95% CI: -4.0 to 5.3) and increased in group 3 participants (adjusted mean difference: 7.9; 95% CI 2.6 to 13.2), compared with the control group. Similarly, the time to feeling recovered was reduced in group 2 (adjusted hazard ratio 1.7; 95% CI: 0.7 to 4.1) but increased in group 3 (adjusted hazard ratio 0.6; 95% CI: 0.2 to 1.4), compared with controls. Of women randomly assigned to take antibiotics alone (controls), one third of respondents reported consuming cranberry products (33.3%, 4/12) (table 4).

An exploratory analysis was conducted to compare the proportion of participants experiencing 'double sickening' between groups at two weeks. Double sickening was defined as a participant selecting the relevant checkbox in the electronic diary to confirm that they felt fully recovered, then subsequently indicating through checkbox responses that they no longer felt fully recovered. Given the short period of time over which this was assessed (2 weeks from enrolment), it is likely that these double sickening episodes represent incompletely treated infection, rather than a new infection. Compared with the control group,



For peer review only - http://bmjopen.bmj.com/site/about/guidelines.xhtml

group 2 participants had a reduced risk of experiencing double sickening (risk ratio 0.6; 95% CI: 0.2 to 2.0), whilst none of the group 3 participants experienced double sickening.

**Table 4** – Secondary outcomes

| Proportion (%), unless otherwise specified | Group 1 (n=15) | Group 2 (n=15 ) | Group 3 (n=16) | Total (n=46 ) |
|---|---|---|---|---|
| **Any of the following treatments used in the past 2 weeks (not including study medication)** | | | | |
| *Cranberry products* | | | | |
| Yes | 4/12 (33.3%) | 2/9 (22.2%) | 2/9 (22.2%) | 8/30 (26.7%) |
| No | 8/12 (66.7%) | 7/9 (77.8%) | 7/9 (77.8%) | 22/30 (73.3%) |
| Missing | 3/15 | 6/15 | 7/16 | 16/46 |
| *Other fruit juice* | | | | |
| Yes | 0/12 (0%) | 0/9 (0%) | 0/9 (0%) | 0/30 (0%) |
| No | 12/12 (100%) | 9/9 (100%) | 9/9 (100%) | 30/30 (100%) |
| Missing | 3/15 | 6/15 | 7/16 | 16/46 |
| *Bicarbonate solution* | | | | |
| Yes | 0/12 (0%) | 0/9 (0%) | 0/9 (0%) | 0/30 (0%) |
| No | 12/12 (100%) | 9/9 (100%) | 9/9 (100%) | 30/30 (100%) |
| Missing | 3/15 | 6/15 | 7/16 | 16/46 |
| *Sodium/potassium citrate (cystitis sachets)* | | | | |
| Yes | 0/12 (0%) | 0/9 (0%) | 1/9 (11.1%) | 1/30 (3.3%) |
| No | 12/12 (100%) | 9/9 (100%) | 8/9 (88.9%) | 29/30 (96.7%) |
| Missing | 3/15 | 6/15 | 7/16 | 16/46 |
| *Uvacin/Uva ursi (bearberry)* | | | | |
| Yes | 0/12 (0%) | 0/9 (0%) | 0/9 (0%) | 0/30 (0%) |
| No | 12/12 (100%) | 9/9 (100%) | 9/9 (100%) | 30/30 (100%) |
| Missing | 3/15 | 6/15 | 7/16 | 16/46 |
| *Pain relief (e.g. paracetamol/Ibuprofen)* | | | | |
| Yes | 5/12 (33.3%) | 1/9 (11.1%) | 5/9 (55.6%) | 11/30 (36.7%) |
| No | 7/12 (58.3%) | 8/9 (88.9%) | 4/9 (44.4%) | 19/30 (63.3%) |



For peer review only - http://bmjopen.bmj.com/site/about/guidelines.xhtml

| | | | | |
|---|---|---|---|---|
| Missing | 3/15 | 6/15 | 7/16 | 16/46 |
| *Other* | | | | |
| Yes | 0/12 (0%) | 0/9 (0%) | 0/9 (0%) | 0/30 (0%) |
| No | 12/12 (100%) | 9/9 (100%) | 9/9 (100%) | 30/30 (100%) |
| Missing | 3/15 | 6/15 | 7/16 | 16/46 |
| *None* | | | | |
| Yes | 5/12 (41.7%) | 6/9 (66.7%) | 3/9 (33.3%) | 14/30 (46.7%) |
| No | 7/12 (58.3%) | 3/9 (33.3%) | 6/9 (66.7%) | 16/30 (53.3%) |
| Missing | 3/15 | 6/15 | 7/16 | 16/46 |
| **Time off paid work or normal activities** | | | | |
| Yes | 5/13 (38.5%) | 2/10 (20%) | 3/9 (33.3%) | 10/32 (31.3%) |
| No. of days, median (IQR) | 1 (1-2) | 5 (2-8) | 2 (1-2) | 2 (1-2) |
| No | 8/13 (61.5%) | 8/10 (80%) | 6/9 (66.7%) | 22/32 (68.8%) |
| Missing | 2/15 | 5/15 | 7/16 | 14/46 |
| **Healthcare professional consulted at GP practice** | | | | |
| Yes | 6/13 (46.2%) | 1/8 (12.5%) | 5/12 (41.7%) | 12/33 (36.4%) |
| No | 7/13 (53.9%) | 7/8 (87.5%) | 7/12 (58.3%) | 21/33 (63.6%) |
| Missing | 2/15 | 7/15 | 4/16 | 13/46 |
| *GP at surgery* | | | | |
| Yes | 3/13 (23.1%) | 0/8 (0%) | 2/12 (16.7%) | 5/33 (15.2%) |
| No. of times, median (IQR) | 1 (1-1) | N/A | 1 (1-1) | 1 (1-1) |
| No | 3/13 (23.1%) | 1/8 (12.5%) | 3/12 (25%) | 7/33 (21.2%) |
| Not applicable | 7/13 (53.9%) | 7/8 (87.5%) | 7/12 (58.3%) | 21/33 (63.6%) |
| Missing | 2/15 | 7/15 | 4/16 | 13/46 |
| *Nurse at surgery* | | | | |
| Yes | 2/13 (15.4%) | 1/8 (12.5%) | 1/12 (8.3%) | 4/33 (12.1%) |
| No. of times, median (IQR) | 1 (1-1) | 2 (2-2) | 1 (1-1) | 1 (1-1.5) |
| No | 4/13 (30.8%) | 0/8 (0%) | 4/12 (33.3%) | 8/33 (24.2%) |
| Not applicable | 7/13 (53.9%) | 7/8 (87.5%) | 7/12 (58.3%) | 21/33 (63.6%) |



For peer review only - http://bmjopen.bmj.com/site/about/guidelines.xhtml

For peer review only

| | | | | |
|---|---|---|---|---|
| Missing | 2/15 | 7/15 | 4/16 | 13/46 |
| *GP at home* | | | | |
| Yes | 0/13 (0%) | 0/8 (0%) | 0/12 (0%) | 0/33 (0%) |
| No. of times, median (IQR) | N/A | N/A | N/A | N/A |
| No | 6/13 (46.2%) | 1/8 (12.5%) | 5/12 (41.7%) | 12/33 (36.4%) |
| Not applicable | 7/13 (53.9%) | 7/8 (87.5%) | 7/12 (58.3%) | 21/33 (63.6%) |
| Missing | 2/15 | 7/15 | 4/16 | 13/46 |
| *OOH doctor* | | | | |
| Yes | 1/13 (7.7%) | 0/8 (0%) | 0/12 (0%) | 1/33 (3.0%) |
| No. of times, median (IQR) | 1 (1-1) | N/A | N/A | 1 (1-1) |
| No | 5/13 (38.5%) | 1/8 (12.5%) | 5/12 (41.7%) | 11/33 (33.3%) |
| Not applicable | 7/13 (53.9%) | 7/18 (87.5%) | 7/12 (58.3%) | 21/33 (63.6%) |
| Missing | 2/15 | 7/15 | 4/16 | 13/46 |
| *Other* | | | | |
| Yes | 0/13 (0%) | 0/8 (0%) | 2/12 (16.7%) – *Pharmacist (1 participant), Phoned GP (1 participant)* | 2/33 (6.1%) |
| No. of times, median (IQR) | N/A | N/A | 1 (1-1) | 1 (1-1) |
| No | 6/13 (46.2%) | 1/8 (12.5%) | 3/12 (25%) | 10/33 (30.3%) |
| Not applicable | 7/13 (53.9%) | 7/8 (87.5%) | 7/12 (58.3%) | 21/33 (63.6%) |
| Missing | 2/15 | 7/15 | 4/16 | 13/46 |
| **Healthcare professional consulted at A&E** | | | | |
| Yes | 0/13 (0%) | 0/10 (0%) | 0/12 (0%) | 0/35 (0%) |
| No | 13/13 (100%) | 10/10 (100%) | 12/12 (100%) | 35/35 (100%) |
| Missing | 2/15 | 5/15 | 4/16 | 11/46 |
| No. of times, median (IQR) | N/A | N/A | N/A | N/A |
| **Seen by a specialist (excluding hospital admission)** | | | | |



For peer review only - http://bmjopen.bmj.com/site/about/guidelines.xhtml

| | | | | |
|---|---|---|---|---|
| Yes | 0/13 (0%) | 0/10 (0%) | 0/12 (0%) | 0/35 (0%) |
| No | 13/13 (100%) | 10/10 (100%) | 12/12 (100%) | 35/35 (100%) |
| Missing | 2/15 | 5/15 | 4/16 | 11/46 |
| No. of times, median (IQR) | N/A | N/A | N/A | N/A |
| **Admitted to hospital** | | | | |
| Yes | 0/13 (0%) | 0/10 (0%) | 0/12 (0%) | 0/35 (0%) |
| No | 13/13 (100%) | 10/10 (100%) | 12/12 (100%) | 35/35 (100%) |
| Missing | 2/15 | 5/15 | 4/16 | 11/46 |
| No. of nights, median (IQR) | N/A | N/A | N/A | N/A |
| **Positive mid-stream urine culture** | | | | |
| Yes | 10/15 (66.7%) | 7/15 (46.7%) | 8/16 (50%) | 25/46 (54.3%) |
| No | 5/15 (33.3%) | 8/15 (53.3%) | 8/16 (50%) | 21/46 (45.7%) |
| Missing | 0/15 | 0/15 | 0/16 | 0/46 |
| **Proportion with a positive mid-stream urine culture** | 54.3% | | | |

All outcomes reported in relation to the two weeks following trial enrolment.

Group 1 – Immediate antibiotics alone (control); Group 2 – Immediate antibiotics and immediate cranberry; Group 3 – Immediate cranberry and delayed antibiotics.

For peer review only

## Harms

Seven adverse events occurred (table 5). Adverse events three and four occurred in the same participant. Two adverse event descriptions sounded like *possible* pyelonephritis (numbers three and seven). However, one of these adverse events occurred in a participant whose Mid-stream urine (MSU) was negative, who reported feeling fully recovered on day 5 of the study and who did not contact a healthcare practitioner up to 28 days after enrolment. The second occurred in a participant who subsequently contacted her GP practice, however, her clinical syndrome was not reported as pyelonephritis by the consulting healthcare practitioner and the participant's symptoms settled with increased fluid intake alone. There were no serious adverse events.



For peer review only - http://bmjopen.bmj.com/site/about/guidelines.xhtml

**Table 5** – List of Adverse Events

| Adverse Event Number | Group | Adverse Event | Outcome | Severity | Relationship to study product | Comments |
|---|---|---|---|---|---|---|
| 1 | Immediate antibiotics alone | Tummy upset that started 3 days after starting Ciprofloxacin (second course of antibiotics) | Resolved | Mild | Not related | Not applicable |
| 2 | Immediate antibiotics alone | Reaction after taking one dose of antibiotic | Resolved | Uncertain | Not related | Not applicable |
| 3 | Immediate antibiotics and immediate cranberry | Suprapubic pain, dysuria, backache, cloudy urine and single vomit | Resolved | Moderate | Possibly related | Possible pyelonephritis from description of symptoms, although not reported as such by healthcare practitioner. MSU positive - E.coli $10^5$ CFU/ml. |
| 4 | Immediate antibiotics and immediate cranberry | Vomiting after second course of Nitrofurantoin started | Resolved | Mild | Possibly related | Vomit after second course of antibiotics started. Antibiotics stopped and participant encouraged to push fluids. Symptoms settled without further antibiotics being prescribed. |
| 5 | Immediate cranberry and delayed antibiotics | Feeling unwell, ache in lower back | Resolved | Moderate | Possibly related | Symptoms unlikely to be related to the study product, as the participant was recruited in the morning and the symptoms started the same day in the afternoon. Participant reported feeling fully recovered on day 2 and did not contact healthcare practitioner up to 28 days. MSU positive > E.coli $10^5$ CFU/ml |
| 6 | Immediate cranberry and delayed antibiotics | Felt sick | Resolved | Mild | Possibly related | Participant mentioned that she was unsure whether the sickness symptom was related to cranberry or her UTI. |
| 7 | Immediate cranberry and delayed antibiotics | General feeling of being unwell, terrible back ache and stomach ache at times. Headache and nausea | Resolved | Moderate | Possibly related | Possible pyelonephritis, however, no growth on MSU, reported feeling fully recovered on day 5, and the participant did not contact a healthcare practitioner up to 28 days. |

**Abbreviations:** MSU: Mid-stream urine; CFU: Colony forming unit

The difference in the proportion of adverse events between groups was compared, although the feasibility trial was not powered to detect differences between groups. At two weeks, the point estimates indicated an increased risk of experiencing an adverse event for participants in group 2 (risk ratio 1.2; 95% CI: 0.1 to 16.7) and group 3 (risk ratio 3.5; 95% CI: 0.4 to 29.4) compared with the control group, although the 95% confidence intervals were wide. At four weeks, the risk of experiencing an adverse event in group 2 was lower than for the control group (risk ratio 0.6; 95% CI: 0.1 to 5.7). The risk remained higher for participants in group 3 compared with controls at four weeks (risk ratio 1.7; 95% CI: 0.3 to 8.8) (Supplementary File 1).

For peer review only

## Discussion

In this open-label, feasibility randomised trial, we recruited beyond target and at a rate faster than predicted. Overall, participants found the trial procedures and interventions acceptable; few reported problems consuming cranberry capsules or using the electronic symptom diary.

<u>Strengths and Weaknesses</u>

To our knowledge, this is the first trial to use cranberry capsules alone and combined with antibiotics as an *acute* treatment for UTI. The trial processes were designed, in discussion with PPI contributors, to be minimally burdensome for participants and recruiters, which may have contributed to the faster than anticipated recruitment, and to recruiting beyond target.

Using a participant-completed diary led to missing data, and in particular, incomplete baseline data. We also collected very limited demographic data. Missing data means that we do not have a full picture of all of the participants' experiences and outcomes. However, insights gained from the trial combined with the qualitative interviews (to be reported elsewhere) should help to optimise future trial design and minimise missing data in a future study.

Some may describe the open-label design as a weakness. However, this was designed to be a pragmatic trial with procedures closely aligned with real-world practice. We were therefore not prescriptive about the antibiotic issued, nor the duration, recognising that clinicians' prescribing is influenced by a host of factors, including a patient's past medical history and previous UTI history. Despite this, we appreciate that there is a lack of standardisation of antibiotic prescribing between groups. Furthermore, participants who missed diary entries



For peer review only - http://bmjopen.bmj.com/site/about/guidelines.xhtml

were able to complete the diary retrospectively; retrospective entries are likely to be less accurate than real-time diary completion.

We did not control participants' consumption of other sources of PAC with Type A linkages, namely blueberry. However, unless participants regularly consumed large amounts of blueberry it is unlikely that this would have had a significant impact on the results. Interestingly, despite advice not to, one third of women in the immediate antibiotics alone group reported consuming cranberry products during the trial. This highlights the challenge of trying to control external influences in a trial, particularly when this is in conflict with a participant's usual behaviour.

Comparison with the existing literature

No study participants developed pyelonephritis. A number of trials of non-antibiotic treatments have reported higher levels of pyelonephritis in participants not assigned to an antibiotic arm.[3 5-7] The absence of cases of pyelonephritis in this trial may well have been due to the small sample size, but may also have resulted from all participants receiving a prescription for antibiotics, whether immediate or delayed antibiotics. In a trial of 382 women with uncomplicated UTIs evaluating the use of ibuprofen and the herbal extract Uva ursi, all participants received delayed antibiotics and there were no cases of pyelonephritis[4].

The proportion of participants lost to follow-up or withdrawing from this feasibility trial (11%) is roughly comparable with analogous trials, which report between 1.3%[17] and 16%[7]. Of note, loss to follow-up is defined differently in different studies. The proportion of participants with a positive urine culture (54.3%) was lower than the proportion reported in some analogous trials (range of 32%[4] to 80%[17]). Of note, this trial used a cut-off threshold for urine culture positivity of $10^5$ CFU/ml, whilst most other trials were aligned with European guidance and used a threshold of greater than $10^2$ or $10^3$CFU/ml.



For peer review only - http://bmjopen.bmj.com/site/about/guidelines.xhtml

Whilst not powered to detect differences between groups, in line with the findings of similar trials, participants in this feasibility trial not receiving immediate antibiotics (group 3) consumed fewer antibiotics but had a higher symptom burden. However, participants in group 3 were less likely to experience double sickening. Interestingly, Gagyor and colleagues[6] found that significantly fewer participants receiving ibuprofen compared with antibiotics experienced UTI recurrence between days 15 to 28 [percentage mean difference -5.3% (95%CI: -10.2% to -0.4%), p = 0.049]. However, the difference between groups did not reach statistical significance when all recurrent UTIs from day 1 to day 28 were assessed [percentage mean difference -2.8% (95%CI: -8.7% to 3.1%), p = 0.41].

A Cochrane review in 2012 found that overall, treatment with cranberry did not significantly reduce *recurrent* UTIs (n=13 studies; risk ratio 0.86; 95% CI: 0.71 to 1.04).[18] This was also the case in a sub-group analysis that focused on women with UTI (n=4 studies; risk ratio 0.74; 95% CI: 0.42 to 1.31). However, the review authors acknowledged that many included studies had high rates of participant withdrawal/loss to follow-up of up to 55%, largely attributed to difficulty adhering to drinking large volumes of cranberry juice on a daily basis. Furthermore, there was a lack of standardisation and poor reporting of the amount of cranberry extract - and specifically of proanthocyanidin (PAC) - in the interventions used. In this feasibility trial, we used cranberry capsules with a standardised amount of PAC, for short-term use in the context of acute UTI. These key differences may have contributed to better adherence of participants to the study intervention.

Implications for clinicians, researchers and policymakers



For peer review only - http://bmjopen.bmj.com/site/about/guidelines.xhtml

467 This feasibility trial was not powered to detect differences between groups, thus, no definite inferences about the effectiveness or safety of cranberry in the context of acute uncomplicated UTI can be made. If cranberry extract is found to be safe and effective in the management of acute UTIs through robust and adequately powered trials, it could represent an important health intervention for this common condition and has the potential to significantly reduce antibiotic consumption.

There is a need for adequately powered, randomised clinical trials to provide definitive evidence of whether cranberry extract is effective in reducing antibiotic use and managing symptoms of acute UTI. Whilst this feasibility trial does not provide evidence of the effectiveness or safety of cranberry extract in the context of acute UTI, the exploratory analyses suggest that it would be worthwhile to explore these as yet unanswered questions through an adequately powered trial. Such trials should use cranberry products with standardised amounts of PAC. Participants should be willing to stop all non-trial cranberry products for the duration of the study; as a minimum, the use of non-trial cranberry products by participants should be captured.

## Conclusion

It is feasible to conduct a randomised trial of similar design to this study to assess whether cranberry extract can help reduce antibiotic use for symptoms of acute, uncomplicated UTI. The exploratory, underpowered analyses suggest that cranberry (alone and combined with antibiotics) *may* have a beneficial impact on certain measures of antibiotic usage and symptoms. These findings support the conduct of further, definitive research in this area.



For peer review only - http://bmjopen.bmj.com/site/about/guidelines.xhtml

## LIST OF ABBREVIATIONS

CFU – Colony Forming Unit

GP – General Practitioner

HRA – Health Research Authority

MHRA - Medicines and Healthcare products Regulatory Agency

NDPCHS - Nuffield Department for Primary Care Health Sciences

PAC – Proanthocyanidin

REDCap - Research Electronic Data Capture

UPEC – Uropathogenic *Escherichia coli*

UTI – Urinary tract infection

## DECLARATIONS

**Contributors:**

OAG was responsible for the research questions, writing the protocol, the design and running of the feasibility trial, data analysis and writing the manuscript. OAG is the guarantor of the manuscript. JA is a senior trial manager who assisted with running the feasibility trial and contributed to the manuscript. NW is a senior statistician who contributed to the protocol and statistical analysis plan, and verified statistical analyses of primary outcomes. CCB, MM, ADH and CJH helped to develop the research questions and study design and contributed to the manuscript. AB was a member of the Trial Management Group and contributed to the



For peer review only - http://bmjopen.bmj.com/site/about/guidelines.xhtml

manuscript. The corresponding author attests that all listed authors meet authorship criteria and that no others meeting the criteria have been omitted.

**Funding:**

This work was supported by the NIHR School for Primary Care Research (Grant reference number: SPCR-2014-10043). Indena S.p.A supplied Redicran capsules to the trial at no cost - they had no other role in the study. OAG's time is funded by the Wellcome Trust (Grant reference number: 203921). CJH received funding support from the NIHR SPCR Evidence Synthesis Working Group [project 390] and the NIHR Oxford BRC. AB receives funding from the NIHR School for Primary Care Research. The funders have no role in study design, manuscript submission, or collection, management, analysis or interpretation of study data. The views are those of the authors and not necessarily those of the NIHR or Department of Health and Social Care, nor the Wellcome Trust. For the purpose of Open Access, the author has applied a CC BY public copyright licence to any Author Accepted Manuscript version arising from this submission.

**Competing interests:**

All authors have completed the ICMJE uniform disclosure form at www.icmje.org/coi_disclosure.pdf and declare: authors had financial support from NIHR SPCR, the Wellcome Trust and the NIHR Oxford BRC for the submitted work; no financial relationships with any organisations that might have an interest in the submitted work in the previous three years; no other relationships or activities that could appear to have influenced the submitted work.

OAG receives funding from the NIHR SPCR and the Wellcome Trust. CCB is; a Senior Investigator of the National Institute of Health Research; Clinical Director of the University



For peer review only - http://bmjopen.bmj.com/site/about/guidelines.xhtml

of Oxford Primary Care and Vaccines Collaborative Clinical Trials Unit; Clinical Director of the NIHR Oxford Community Medtech and Invitro diagnostics Cooperative, and; salaried general practitioner for the Cwm Taf Morgannwg University Health Board. He has received funding from many public funding bodies for primary care research related to the management of common infections. He received payment for contributing to Advisory Boards for Pfizer in 2019, Roche Diagnostics in 2020, and for contributing to an Advisory Board for Janssen Pharmaceuticals about Respiratory Syncytial Virus treatment and vaccination from Janssen Pharmaceuticals in 2020, and holds an unrestricted grant form Janssen Pharmaceuticals for contributing to research on Respiratory Syncytial Virus. CJH reports he has received expenses and fees for his media work. He is Director of the CEBM at the University of Oxford, and Editor in Chief of BMJ Evidence-Based Medicine and an NIHR Senior Investigator. JA, NW, MM, ADH and AB have no Conflict of Interests to declare.

**Figure Legend:**

Figure 1 – CONSORT diagram showing the flow of trial participants.

**Protocol:**

The protocol has been published and is freely available:

Gbinigie, O., Allen, J., Boylan, A. *et al.* Does cranberry extract reduce antibiotic use for symptoms of acute uncomplicated urinary tract infections (CUTI)? Protocol for a feasibility study. *Trials* **20,** 767 (2019). https://doi.org/10.1186/s13063-019-3860-z



For peer review only - http://bmjopen.bmj.com/site/about/guidelines.xhtml

**Ethics Approval:**

Informed consent was obtained from all participants prior to entry into the study. In accordance with the Declaration of Helsinki, the CUTI study was approved by the South Central Oxford B Ethics Committee (REC reference: 18/SC/0673) and the Health Research Authority (IRAS Project ID: 249672).

**Transparency Statement:**

The lead author (the manuscript's guarantor) affirms that the manuscript is an honest, accurate, and transparent account of the study being reported; that no important aspects of the study have been omitted; and that any discrepancies from the study as originally planned (and, if relevant, registered) have been explained.

**Acknowledgements:**

The authors would like to thank all study participants, participating GP practices and staff in Oxford University’s Primary Care Clinical Trials Unit who were responsible for electronic database development (Ms Rui Zhao and Mr Luis Castello).

The authors would also like to thank members of the Trial Steering Committee (TSC): Dr. Akke Vellinga (TSC Chair and epidemiologist); Ms. Julia Hamer-Hunt (PPI representative); Dr. Bernard Gudgin (PPI representative); Dr. Merlin Willcox (academic clinical lecturer and GP); Dr. Emma Thomas-Jones (research fellow and senior trials manager) and Dr. Kathy Hughes (clinical lecturer and GP).

We thank Dr Sarah Tonkin-Crine for helpful feedback on the manuscript, and PPI contributors for their involvement with the trial.



For peer review only - http://bmjopen.bmj.com/site/about/guidelines.xhtml

**Data sharing statement:**

Relevant anonymised data are available upon reasonable request made to the corresponding author.

**Patient consent for publication:**

Not applicable – no participant identifiable information included

For peer review only

## REFERENCES

1. Butler CC, Hillier S, Roberts Z, et al. Antibiotic-resistant infections in primary care are symptomatic for longer and increase workload: outcomes for patients with E. coli UTIs. 2006;56(530):686-92.
2. Little P, Merriman R, Turner S, et al. Presentation, pattern, and natural course of severe symptoms, and role of antibiotics and antibiotic resistance among patients presenting with suspected uncomplicated urinary tract infection in primary care: observational study. *BMJ* 2010;340:b5633.
3. Kronenberg A, Bütikofer L, Odutayo A, et al. Symptomatic treatment of uncomplicated lower urinary tract infections in the ambulatory setting: randomised, double blind trial. *bmj* 2017;359:j4784.
4. Moore M, Trill J, Simpson C, et al. Uva-ursi extract and ibuprofen as alternative treatments for uncomplicated urinary tract infection in women (ATAFUTI): a factorial randomized trial. *Clinical Microbiology and Infection* 2019;25(8):973-80.



For peer review only - http://bmjopen.bmj.com/site/about/guidelines.xhtml

5. Wagenlehner FM, Abramov-Sommariva D, Höller M, et al. Non-antibiotic herbal therapy (BNO 1045) versus antibiotic therapy (Fosfomycin Trometamol) for the treatment of acute lower uncomplicated urinary tract infections in women: a double-blind, parallel-group, randomized, multicentre, non-inferiority phase III trial. *J Urologia internationalis* 2018;101(3):327-36.
6. Gágyor I, Bleidorn J, Kochen MM, et al. Ibuprofen versus fosfomycin for uncomplicated urinary tract infection in women: randomised controlled trial. *bmj* 2015;351:h6544.
7. Vik I, Bollestad M, Grude N, et al. Ibuprofen versus pivmecillinam for uncomplicated urinary tract infection in women—A double-blind, randomized non-inferiority trial. *PLoS medicine* 2018;15(5):e1002569.
8. Butler CC, Hawking MK, Quigley A, et al. Incidence, severity, help seeking, and management of uncomplicated urinary tract infection: a population-based survey. *Br J Gen Pract* 2015;65(639):e702-e07.
9. Howell AB, Botto H, Combescure C, et al. Dosage effect on uropathogenic Escherichia coli anti-adhesion activity in urine following consumption of cranberry powder standardized for proanthocyanidin content: a multicentric randomized double blind study. *BMC infectious diseases* 2010;10(1):94.
10. Howell AB, Reed JD, Krueger CG, et al. A-type cranberry proanthocyanidins and uropathogenic bacterial anti-adhesion activity. *Phytochemistry* 2005;66(18):2281-91.
11. Gbinigie O, Allen J, Boylan A-M, et al. Does cranberry extract reduce antibiotic use for symptoms of acute uncomplicated urinary tract infections (CUTI)? Protocol for a feasibility study. *Trials* 2019;20(1):1-9.
12. Watson L, Little P, Moore M, et al. Validation study of a diary for use in acute lower respiratory tract infection. *Family Practice* 2001;18(5):553-54.
13. National Institute for Health and Care Excellence. Urinary tract infection (lower): antimicrobial prescribing. NICE guideline [NG109] 2018 [Available from: https://www.nice.org.uk/guidance/ng109.
14. Foden N, Burgess C, Shepherd K, et al. A guide to the management of acute rhinosinusitis in primary care management strategy based on best evidence and recent European guidelines. 2013;63(616):611-13.
15. Julious SA. Sample size of 12 per group rule of thumb for a pilot study. *J Pharmaceutical Statistics: The Journal of Applied Statistics in the Pharmaceutical Industry* 2005;4(4):287-91.
16. Sim J, Lewis M. The size of a pilot study for a clinical trial should be calculated in relation to considerations of precision and efficiency. *Journal of clinical epidemiology* 2012;65(3):301-08.
17. Bleidorn J, Gágyor I, Kochen MM, et al. Symptomatic treatment (ibuprofen) or antibiotics (ciprofloxacin) for uncomplicated urinary tract infection?-results of a randomized controlled pilot trial. *BMC medicine* 2010;8(1):30.
18. Jepson RG, Williams G, Craig JC. Cranberries for preventing urinary tract infections. *The Cochrane Library* 2012



For peer review only - http://bmjopen.bmj.com/site/about/guidelines.xhtml

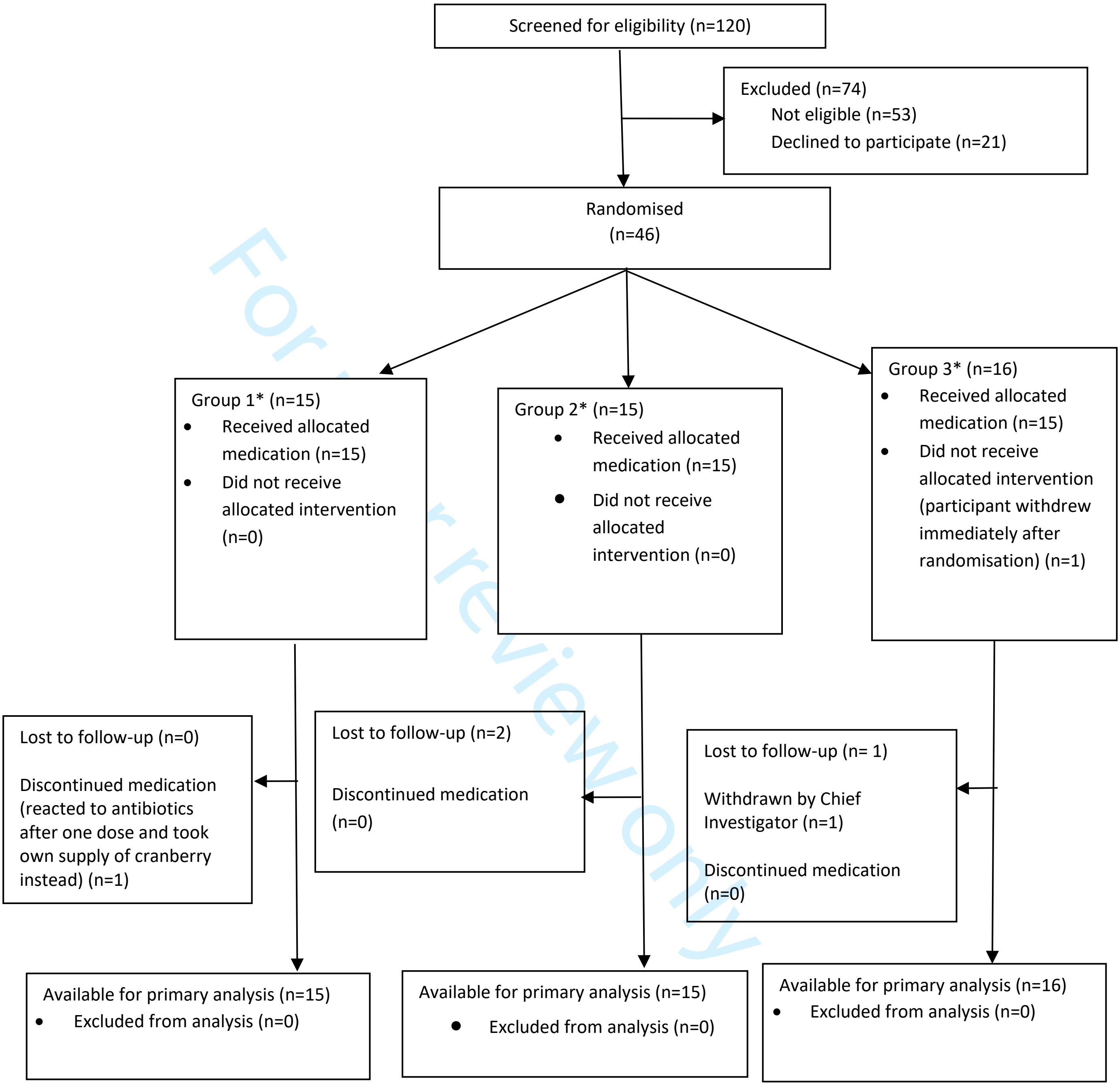


**Figure 1** – CONSORT diagram showing the flow of trial participants.
*Group 1 – Immediate antibiotics alone (control); Group 2 – Immediate antibiotics and immediate cranberry capsules; Group 3 – Immediate cranberry capsules and delayed antibiotics

For peer review only - http://bmjopen.bmj.com/site/about/guidelines.xhtml

Supplementary Figures

**Kaplan Meier survival plot showing the duration of symptoms rated moderately bad or worse by group**

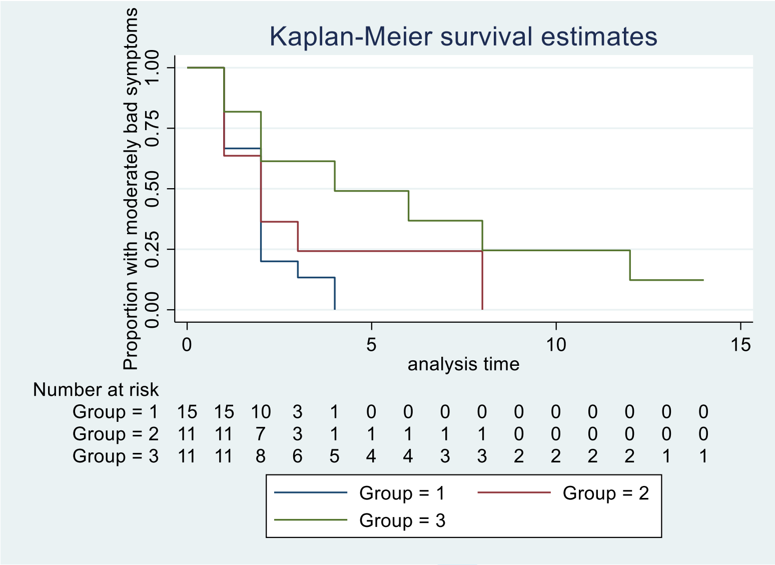


**Key:** Group 1: Immediate Antibiotics alone; Group 2: Immediate Antibiotics and Immediate Cranberry; Group 3: Immediate Cranberry and Delayed Antibiotics

**Kaplan Meier survival plot showing the time to feeling fully recovered by group**

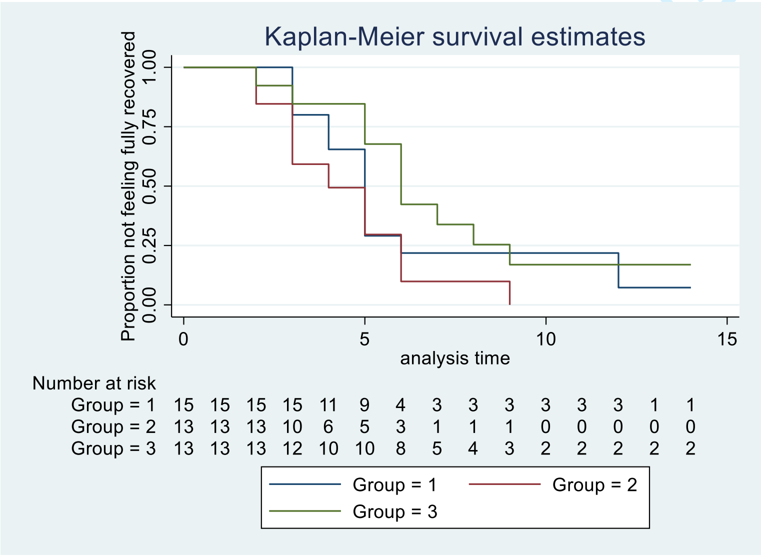


**Key:** Group 1: Immediate Antibiotics alone; Group 2: Immediate Antibiotics and Immediate Cranberry; Group 3: Immediate Cranberry and Delayed Antibiotics

For peer review only

**Supplementary file 1** – Estimate of the difference in proportions of adverse events between groups

| | Proportion (%) of participants experiencing an adverse event at 2 weeks [risk ratio (95% CI)] | Proportion (%) of participants experiencing an adverse event at 4 weeks [risk ratio (95% CI)] |
|---|---|---|
| **Group 1** (Control) n=15 | 1/15 (6.7%)<br><br>Missing: 0/15 | 2/15 (13.3%)<br><br>Missing: 0/15 |
| **Group 2** n=15 | 1/13 (7.7%)<br>[1.2 (0.1 to 16.7)]<br><br>Missing: 2/15 | 1/13 (7.7%)<br>[0.6 (0.1 to 5.7)]<br><br>Missing: 2/15 |
| **Group 3** n=16 | 3/13 (23.1%)<br>[3.5 (0.4 to 29.4)]<br><br>Missing: 3/16 | 3/13 (23.1%)<br>[1.7 (0.3 to 8.8 )]<br><br>Missing: 3/16 |

Group 1 – Immediate antibiotics alone (control); Group 2 – Immediate antibiotics and immediate cranberry; Group 3 – Immediate cranberry and delayed antibiotics. Group 2 and group 3 were compared with Group 1 (controls).

For peer review only

# CONSORT 2010 checklist of information to include when reporting a pilot or feasibility trial*

| Section/Topic | Item No | Checklist item | Reported on page No |
|---|---|---|---|
| **Title and abstract** | | | |
| | 1a | Identification as a pilot or feasibility randomised trial in the title | 1 |
| | 1b | Structured summary of pilot trial design, methods, results, and conclusions (for specific guidance see CONSORT abstract extension for pilot trials) | 2-3 |
| **Introduction** | | | |
| Background and objectives | 2a | Scientific background and explanation of rationale for future definitive trial, and reasons for randomised pilot trial | 4 |
| | 2b | Specific objectives or research questions for pilot trial | 4 |
| **Methods** | | | |
| Trial design | 3a | Description of pilot trial design (such as parallel, factorial) including allocation ratio | 5 |
| | 3b | Important changes to methods after pilot trial commencement (such as eligibility criteria), with reasons | N/A |
| Participants | 4a | Eligibility criteria for participants | 5 |
| | 4b | Settings and locations where the data were collected | 5 |
| | 4c | How participants were identified and consented | 5 |
| Interventions | 5 | The interventions for each group with sufficient details to allow replication, including how and when they were actually administered | 6 |
| Outcomes | 6a | Completely defined prespecified assessments or measurements to address each pilot trial objective specified in 2b, including how and when they were assessed | 6-7 and protocol |
| | 6b | Any changes to pilot trial assessments or measurements after the pilot trial commenced, with reasons | 7 |
| | 6c | If applicable, prespecified criteria used to judge whether, or how, to proceed with future definitive trial | N/A |
| Sample size | 7a | Rationale for numbers in the pilot trial | 7 |
| | 7b | When applicable, explanation of any interim analyses and stopping guidelines | N/A |
| Randomisation: | | | |
| Sequence generation | 8a | Method used to generate the random allocation sequence | 7 |
| | 8b | Type of randomisation(s); details of any restriction (such as blocking and block size) | 7 |
| Allocation concealment mechanism | 9 | Mechanism used to implement the random allocation sequence (such as sequentially numbered containers), describing any steps taken to conceal the sequence until interventions were assigned | 7 |

For peer review only

| | | | |
|---|---|---|---|
| Implementation | 10 | Who generated the random allocation sequence, who enrolled participants, and who assigned participants to interventions | 7 |
| Blinding | 11a | If done, who was blinded after assignment to interventions (for example, participants, care providers, those assessing outcomes) and how | N/A |
| | 11b | If relevant, description of the similarity of interventions | N/A |
| Statistical methods | 12 | Methods used to address each pilot trial objective whether qualitative or quantitative | 8-9 |
| **Results** | | | |
| Participant flow (a diagram is strongly recommended) | 13a | For each group, the numbers of participants who were approached and/or assessed for eligibility, randomly assigned, received intended treatment, and were assessed for each objective | Figure 1 |
| | 13b | For each group, losses and exclusions after randomisation, together with reasons | Figure 1 |
| Recruitment | 14a | Dates defining the periods of recruitment and follow-up | 14 |
| | 14b | Why the pilot trial ended or was stopped | 14 |
| Baseline data | 15 | A table showing baseline demographic and clinical characteristics for each group | Table 1 |
| Numbers analysed | 16 | For each objective, number of participants (denominator) included in each analysis. If relevant, these numbers should be by randomised group | Figure 1 |
| Outcomes and estimation | 17 | For each objective, results including expressions of uncertainty (such as 95% confidence interval) for any estimates. If relevant, these results should be by randomised group | Table 3 |
| Ancillary analyses | 18 | Results of any other analyses performed that could be used to inform the future definitive trial | 24 and Table 3 |
| Harms | 19 | All important harms or unintended effects in each group (for specific guidance see CONSORT for harms) | 24 and Table 5 |
| | 19a | If relevant, other important unintended consequences | N/A |
| **Discussion** | | | |
| Limitations | 20 | Pilot trial limitations, addressing sources of potential bias and remaining uncertainty about feasibility | 26-27 |
| Generalisability | 21 | Generalisability (applicability) of pilot trial methods and findings to future definitive trial and other studies | 28-29 |
| Interpretation | 22 | Interpretation consistent with pilot trial objectives and findings, balancing potential benefits and harms, and considering other relevant evidence | 27-28 |
| | 22a | Implications for progression from pilot to future definitive trial, including any proposed amendments | 28 |
| **Other information** | | | |
| Registration | 23 | Registration number for pilot trial and name of trial registry | 3 |
| Protocol | 24 | Where the pilot trial protocol can be accessed, if available | 31-32 |
| Funding | 25 | Sources of funding and other support (such as supply of drugs), role of funders | 31 |

For peer review only

| | | | |
|---|---|---|---|
| | 26 | Ethical approval or approval by research review committee, confirmed with reference number | 32-33 |

Citation: Eldridge SM, Chan CL, Campbell MJ, Bond CM, Hopewell S, Thabane L, et al. CONSORT 2010 statement: extension to randomised pilot and feasibility trials. BMJ. 2016;355.
*We strongly recommend reading this statement in conjunction with the CONSORT 2010, extension to randomised pilot and feasibility trials, Explanation and Elaboration for important clarifications on all the items. If relevant, we also recommend reading CONSORT extensions for cluster randomised trials, non-inferiority and equivalence trials, non-pharmacological treatments, herbal interventions, and pragmatic trials. Additional extensions are forthcoming: for those and for up to date references relevant to this checklist, see www.consort-statement.org.

For peer review only
